# Gut microbiota production of trimethyl-5-aminovaleric acid reduces fatty acid oxidation and accelerates cardiac hypertrophy

**DOI:** 10.1038/s41467-022-29060-7

**Published:** 2022-04-01

**Authors:** Mingming Zhao, Haoran Wei, Chenze Li, Rui Zhan, Changjie Liu, Jianing Gao, Yaodong Yi, Xiao Cui, Wenxin Shan, Liang Ji, Bing Pan, Si Cheng, Moshi Song, Haipeng Sun, Huidi Jiang, Jun Cai, Minerva T. Garcia-Barrio, Y. Eugene Chen, Xiangbao Meng, Erdan Dong, Dao Wen Wang, Lemin Zheng

**Affiliations:** 1 Department of Cardiology and Institute of Vascular Medicine, Peking University Third Hospital, Beijing, China; 2 The Institute of Cardiovascular Sciences, School of Basic Medical Sciences, NHC Key Laboratory of Cardiovascular Molecular Biology and Regulatory Peptides; Key Laboratory of Molecular Cardiovascular Science, Ministry of Education; Beijing Key Laboratory of Cardiovascular Receptors Research, Peking University, Beijing, 100191 China; 3 Division of Cardiology, Department of Internal Medicine and Hubei Key Laboratory of Genetics and Molecular Mechanisms of Cardiological Disorders, Tongji Hospital, Tongji Medical College, Huazhong University of Science and Technology, Wuhan, 430030 China; 4 Department of Cardiology, Zhongnan Hospital of Wuhan University, Wuhan, 430071 China; 5 Laboratory of Pharmaceutical Analysis and Drug Metabolism, College of Pharmaceutical Sciences, Zhejiang University, Hangzhou, 310058 China; 6 Department of Cardiology, The First Affiliated Hospital, College of Medicine, Zhejiang University, Hangzhou, China; 7 Beijing Tiantan Hospital, China National Clinical Research Center for Neurological Diseases, Advanced Innovation Center for Human Brain Protection, The Capital Medical University, Beijing, 100050 China; 8 State Key Laboratory of Membrane Biology, Institute of Zoology, Chinese Academy of Sciences, Beijing, 100101 China; 9 Department of Pathophysiology, Key Laboratory of Cell Differentiation and Apoptosis of Chinese Ministry of Education, Shanghai Jiao Tong University School of Medicine, Shanghai, 200025 China; 10 Fuwai Hospital, State Key Laboratory of Cardiovascular Diseases, National Center for Cardiovascular Diseases, Chinese Academy of Medical Sciences and Peking Union Medical College, Beijing, China; 11 Cardiovascular Center, Department of Internal Medicine, University of Michigan Medical Center, Ann Arbor, MI 48109 USA; 12 State Key Laboratory of Natural and Biomimetic Drugs, School of Pharmaceutical Sciences, Peking University, Beijing, 100191 China

**Keywords:** Cardiology, Metabolic disorders, Microbiome

## Abstract

Numerous studies found intestinal microbiota alterations which are thought to affect the development of various diseases through the production of gut-derived metabolites. However, the specific metabolites and their pathophysiological contribution to cardiac hypertrophy or heart failure progression still remain unclear. N,N,N-trimethyl-5-aminovaleric acid (TMAVA), derived from trimethyllysine through the gut microbiota, was elevated with gradually increased risk of cardiac mortality and transplantation in a prospective heart failure cohort (*n* = 1647). TMAVA treatment aggravated cardiac hypertrophy and dysfunction in high-fat diet-fed mice. Decreased fatty acid oxidation (FAO) is a hallmark of metabolic reprogramming in the diseased heart and contributes to impaired myocardial energetics and contractile dysfunction. Proteomics uncovered that TMAVA disturbed cardiac energy metabolism, leading to inhibition of FAO and myocardial lipid accumulation. TMAVA treatment altered mitochondrial ultrastructure, respiration and FAO and inhibited carnitine metabolism. Mice with γ-butyrobetaine hydroxylase (BBOX) deficiency displayed a similar cardiac hypertrophy phenotype, indicating that TMAVA functions through BBOX. Finally, exogenous carnitine supplementation reversed TMAVA induced cardiac hypertrophy. These data suggest that the gut microbiota-derived TMAVA is a key determinant for the development of cardiac hypertrophy through inhibition of carnitine synthesis and subsequent FAO.

## Abstract (web summary)

Intestinal microbiota alterations may affect heart function through the production of gut-derived metabolites. Here the authors found that gut microbiota-derived TMAVA is a key determinant for the development of cardiac hypertrophy through inhibition of carnitine synthesis and subsequent fatty acid oxidation.

## Introduction

Heart failure (HF) is a global problem, with an estimated 38 million patients with this diagnosis worldwide^1^. Myocardial hypertrophy is an early milestone during the clinical course of HF and an important risk factor for subsequent cardiac morbidity and mortality^2^. Increased efforts to enhance the understanding of the pathobiology of heart hypertrophy and to develop approaches for preventing cardiac hypertrophy and failure are imperative^3^. Metabolites, the chemical entities that are transformed during metabolism, provide a functional readout of cellular biochemistry. Metabolomics, by performing global metabolite profiling, offers a window for interrogating how mechanistic biochemistry relates to cellular phenotype^4^. Therefore, it provides a tool for discovering new metabolites and advancing the characterization of HF.

The heart is an energy-demanding organ relying on fatty acid (FA) and glucose oxidation. Long-chain FAs contribute up to 70% of the energy required by an adult heart to function under normal physiological conditions^5^. A failing heart usually shows impaired transcription of key enzymes involved in FA metabolism^6,7^. Consequently, the heart switches to utilize glucose as the main fuel. Nevertheless, whether this substrate utilization shift is adaptive or maladaptive remains controversial. Several preclinical and clinical studies showed beneficial effects by inhibiting various steps of FA oxidation (FAO) in animal and human subjects with cardiac hypertrophy and HF^8^. Meanwhile, others reported adverse effects of inhibiting FAO in animal studies. This occurs with carnitine palmitoyltransferase-1β deficiency^9^ and peroxisome proliferator-activated receptor-γ coactivator-1β (PGC-1β) deficiency^10^. Similarly, heart dysfunction is found when mice with heart-specific glucose transporter 1 (GLUT1) overexpression are placed on an HFD^11^.

Further evidence suggests that heart dysfunction is due to excess accumulation of lipid in cardiomyocytes, termed lipotoxic cardiomyopathy or fatty heart^11–13^. Increased uptake of circulating free FA (FFA) or lipoprotein-derived lipids occurring with transgenic expression of lipoprotein lipase (LpL)^14^ leads to reduced cardiac function. Transgenic mice with cardiomyocyte-specific expression of FA transport protein 1^15^ and acyl CoA synthetase 1^16^ are thought to have increased FFA uptake or trapping in the heart, which leads to HF. Peroxisome proliferator-activated receptor (PPAR) transcription factors drive FA oxidation; however, the increased lipoprotein-lipid uptake in PPARα transgenic mice^17^ exceeds the increased FA oxidation found in this model. It is argued that patients with type 2 diabetes, metabolic syndrome, and obesity accumulate excess intramyocardial lipid and exhibit decreased systolic or diastolic function^18^. Diabetic cardiomyopathy, as a major complication, is the leading cause of morbidity and mortality for diabetic patients. Epidemiological studies have demonstrated that diabetic people have a 2- to 5-fold increase in the risk of developing HF compared with age-matched healthy subjects after adjusting for age, blood pressure, weight, cholesterol level, and coronary artery disease^19,20^. The underlying molecular mechanisms could be either increased lipid uptake or impaired mitochondrial oxidative function leading to accumulation of TGs and toxic lipid species.

In this study, we observed that N,N,N-trimethyl-5-aminovaleric acid (TMAVA), is associated with a gradually increased risk of adverse clinical outcomes in higher TMAVA levels using a prospective HF cohort. Mice treated with TMAVA show aggravated cardiac hypertrophy and dysfunction in a high-fat diet (HFD). Mechanistically, carnitine biosynthesis and uptake are inhibited by TMAVA, leading to FFA oxidation inhibition and myocardial lipid accumulation and toxicity. Mitochondria ultrastructure and function are also altered with TMAVA treatment. BBOX-deficient mice display a similar cardiac hypertrophy phenotype which indicates that TMAVA functions through the BBOX pathway. Finally, exogenous carnitine supplementation reverses TMAVA induced cardiac hypertrophy. TMAVA could be a potential therapeutic target for cardiac hypertrophy related to gut microbiota.

## Results

### A higher TMAVA level is associated with an elevated HF risk

**Fig. 1:**
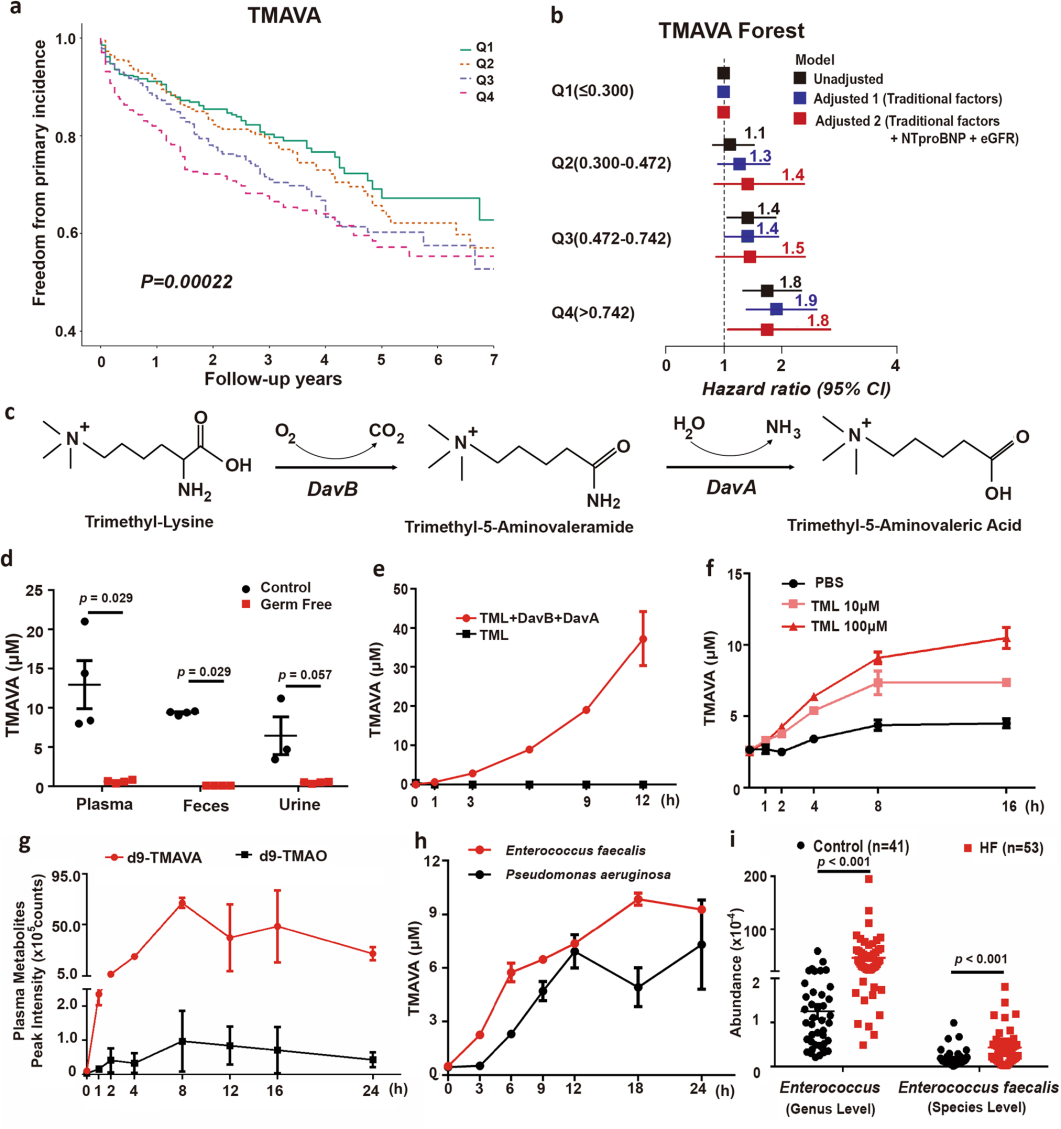
Elevated plasma TMAVA level is associated with incident cardiac death and transplantation risk, independent of traditional risk factors. **a** Kaplan–Meier estimate of 7-year risk for cardiac death and transplantation ranked by TMAVA quartiles in the cohort (*N* = 1647). **b** Forest plots indicate the HR (95% CI) for incident (7-year) risk for cardiac death and transplantation according to TMAVA quartiles. HR (unadjusted, black squares) and multivariate Cox model 1 adjusted (blue squares; adjusted for age, sex, smoking, SBP, diabetes, high-density lipoprotein [HDL], low-density lipoprotein [LDL]), or model 2 adjusted (red squares, adjusted for model 1 plus eGFR and NTproBNP). The 95% confidence interval is indicated by the line length. HR hazard ratio. **c** Schema for the production of TMAVA from TML by the DavB and DavA coupled system. **d** TMAVA levels were significantly lower in plasma, feces, and urine of germ-free mice (*n* = 4). **e** Production of TMAVA from TML by the DavB and DavA coupled system in vitro (*n* = 3). **f** Feces from conventionally raised mice were co-incubated with different TML concentrations and TMAVA levels were measured (*n* = 3). **g** Mice were challenged with d9-TML. Post-challenge measurement of d9-TMAVA and d9-TMAO was performed in serial venous blood draws at the indicated times by stable isotope dilution LC–MS (*n* = 2). **h** *Enterococcus faecalis* and *Pseudomonas aeruginosa* were co-incubated with 1 mM TML and TMAVA levels were measured at the indicated time points (*n* = 2 and 3). **i** Abundance (percentage of the whole microbiota) *of Enterococcus* at the genus level *and E. faecalis* at the species level were analyzed in patients using metagenomics sequencing. Statistical significance was evaluated by a two-tailed nonparametric Mann–Whitney test [(**d**) and (**i**)]. (*P < 0.05, **P < 0.01, ***P < 0.001). Data are expressed as mean ± SEM. Source data are provided as a Source Data file.

During the untargeted metabolomics study previously published^21^, we found that TMAVA was significantly and consistently elevated in subjects with hypertension, a known driver of cardiac hypertrophy (Supplementary Fig. 1a). In an 84-month follow-up of the cohort containing 1647 patients (Supplementary Table 2), primary outcomes (including cardiac death and transplantation) occurred in 387 patients. When the cohort was divided into four quartiles based on TMAVA levels (Q1 ≤ 0.300; Q2 = 0.300–0.472; Q3 = 0.472–0.742; Q4 > 0.742), patients in the highest quartile (Q4) had a significantly elevated risk of cardiac death and transplantation when compared with the lowest quartile with a hazards ratio of 1.76 (95% CI: 1.34–2.34; *P* < 0.001). The Kaplan–Meier analysis also showed a significant increase in mortality risk associated with TMAVA levels (*P* < 0.001) (Fig. 1a). Moreover, an elevated TMAVA level (Q4) is also an independent predictor of 7-year cardiac death and transplantation risk even after adjustments for traditional cardiac risk factors (age, sex, smoking, SBP, diabetes, HDL, and LDL) (adjusted model 1) (hazards ratio [HR], 1.91; 95% CI: 1.39–2.62; *P* < 0.001) and eGFR with NTproBNP levels (adjusted model 2) (HR, 1.75; 95% CI: 1.06–2.86; *P* = 0.027) (Fig. 1b).

Recently, we found that the gut microbiota is obligatory for endogenous TMAVA generation^21^. TMAVA could be produced from TML through the gut microbiota with the enzymes lysine 2-monooxygenase (DavB) and 5-aminovaleramidase (DavA) (Fig. 1c). Germ-free mice displayed trace TMAVA content in plasma, feces, and urine (Fig. 1d). Recent studies found that TML is associated with incident (3-year) major adverse cardiovascular event risks and incident (5-year) mortality risk independently^22,23^. Therefore, recombinant DavB and DavA proteins purified from *Escherichia coli* strains expressing the *davB* and *davA* genes were used to test the production of TMAVA from TML. When the purified DavB and DavA proteins were incubated with TML, TMAVA was produced time-dependently in vitro (Fig. 1e). Additionally, when feces from conventionally raised mice were co-incubated with different TML concentrations, TMAVA levels were also increased in a time and concentration-dependent fashion (Fig. 1f) while sterilized feces failed to produce TMAVA (Supplementary Fig. 1b). Gavage of d9-TML time-dependently increased the plasma levels of d9-TMAVA in mice (Fig. 1g), which indicates a direct precursor/product relationship. A previous study reported that TMAO can be generated from TML^22^. In our study, we found that plasma levels of d9-TMAO rose only minimally following the gavage of TML. By comparison, d9-TMAVA displayed markedly (50- to 100-fold) higher peak levels than those of d9-TMAO (Fig. 1g), and enhanced d9-TMAVA production was impaired when d9-TML was gavaged into germ-free mice or injected intraperitoneal in conventional mice (Supplementary Fig. 1c). These results directly demonstrate that oral TML can preferentially serve as a precursor for TMAVA rather than TMAO in vivo. *Enterococcus faecalis* and *Pseudomonas aeruginosa* showed sequences with 80% identity with *davB* and *davA* in a Megablast search of 36,464 bacteria genomes downloaded from the NCBI. When these bacteria were incubated with 1 mM TML, TMAVA was generated in a time-dependent manner (Fig. 1h). Collectively, these results indicate that TML serves as a precursor for gut microbiota-dependent formation of TMAVA. More importantly, in an independent cohort consisting of 53 HF and 41 controls, metagenomics sequencing was performed. The clinical characteristics of the subjects and the detailed methods for metagenomics were published elsewhere^24^. We found that the genus level of *Enterococcus* was elevated significantly and the species of *E. faecalis* was also increased in patients with HF. The *Pseudomonas* genus level and *P. aeruginosa* species level showed an increased trend without a statistical difference (Fig. 1i, Supplementary Fig. 1d).

### TMAVA levels predict HF risk in various major chronic disease subgroups and elevated plasma TMAVA level is associated with incident cardiac death and transplantation risk, independent of traditional risk factors and TML

**Fig. 2:**
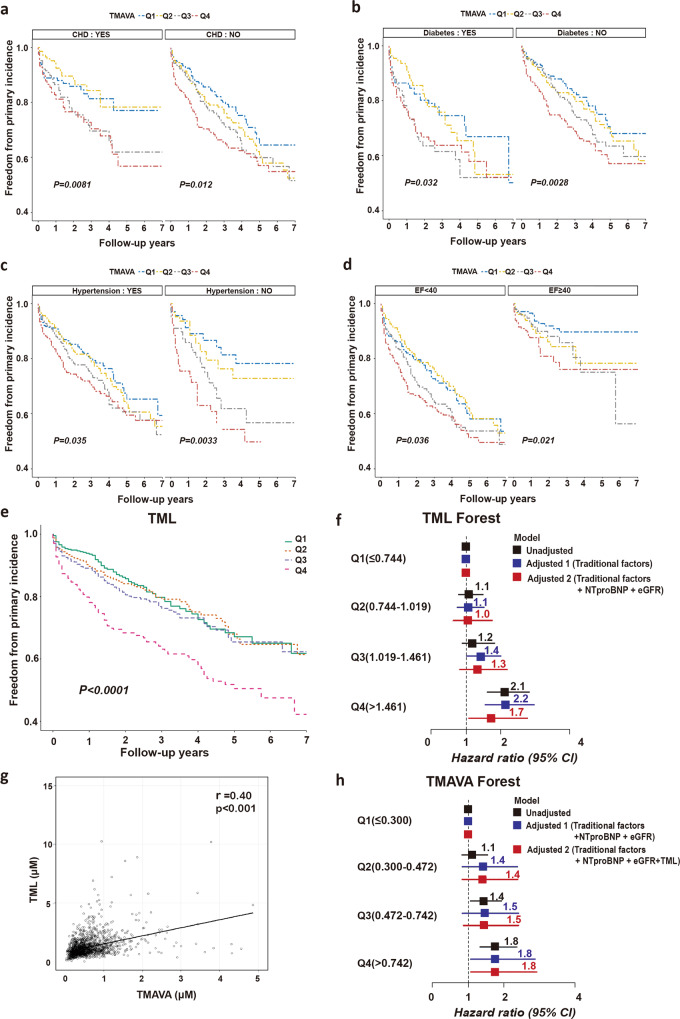
TMAVA levels predict heart failure risk in various chronic disease subgroups and elevated plasma TMAVA level is associated with incident cardiac death and transplantation risk, independent of traditional risk factors and TML. **a**–**d** Kaplan-Meier estimate of 7-year risk for cardiac death and transplantation ranked by TMAVA quartiles in subgroups according to CHD (**a**), diabetes (**b**), hypertension (**c**), and EF (**d**) in the cohort (*N* = 1647). CHD coronary heart disease. **e** Kaplan–Meier estimate of 7-year risk for cardiac death and transplantation ranked by TML quartiles in the cohort (*N* = 1647). **f** Forest plots indicate the HR (95% CI) for incident (5-year) risk for cardiac death and transplantation according to TML quartiles. HR (unadjusted, black squares) and multivariate Cox model 1 adjusted (blue squares; adjusted for age, sex, smoking, SBP, diabetes, high-density lipoprotein [HDL], low-density lipoprotein [LDL]), or model 2 adjusted (red squares, adjusted for model 1 plus eGFR and NTproBNP). The 95% confidence interval is indicated by the line length. HR hazard ratio. **g** Correlation between plasma levels of TMAVA and TML in the validation cohort (*N* = 1647). **h** Forest plots indicate the HR (95% CI) for incident (7-year) risk for cardiac death and transplantation according to TMAVA quartiles. HR (unadjusted, black squares) and multivariate Cox model 1 adjusted (blue squares; adjusted for age, sex, smoking, SBP, diabetes, high-density lipoprotein [HDL], low-density lipoprotein [LDL], NTproBNP, and eGFR), or model 2 adjusted (red squares, adjusted for model 1 plus TML). The 95% confidence interval is indicated by the line length. HR hazard ratio. Source data are provided as a Source Data file.

In order to assess the association between TMAVA and mortality risk among patients with various chronic diseases (84-month follow of a cohort of 1647 patients, and Supplementary Table 2), we analyzed patients with or without coronary heart disease (*n* = 642, 39.0%) and diabetes mellitus (*n* = 493, 29.9%) (Fig. 2a, b). Notably, statistical significance (*P* < 0.05) was also found in patients with coronary heart disease (*P* = 0.008) (Fig. 2a) and diabetes mellitus (*P* = 0.032) (Fig. 2b), with higher levels of TMAVA related to increased adverse outcome risk in those subsets. In this cohort, patients with hypertension (*n* = 1359, 82.5%) also displayed an increased mortality risk with higher TMAVA levels (Fig. 2c). Additionally, 45% of the cohort had ejection fraction (EF) of no less than 40% and 50.3% presented reduced EF (<40%). The remaining participants (4.7%) were not assessed. Figure 2d shows that, among the patients within the two subgroups defined by EF, both groups of patients presented significantly higher mortality risk in association with elevated TMAVA levels. Interaction analysis among the subgroups (CHD or no CHD, diabetes or no diabetes, low or high EF, hypertension or no hypertension) found that there is a significant subgroup interaction between adverse outcomes and hypertension or EF (Supplementary Table 3).

It was reported that the levels of plasma TML, shown here to be the precursor of TMAVA (Fig. 1f–h), is independently associated with incident (3-year) major adverse cardiovascular event risks and incident (5-year) mortality risk^22,23^. In our cohort, Kaplan–Meier analysis of plasma TML showed a gradually increased mortality risk accompanied by graded TML levels, which becomes especially apparent in the higher levels (*P* < 0.0001) (Fig. 2e). We also found that as compared with subjects in the lowest quartile of TML levels, patients in the highest quartile (Q4) presented a significantly increased risk of incident cardiac death and transplantation (HR, 2.14; 95% CI: 1.62–2.83; *P* < 0.001). Moreover, elevated TML (Q4) levels remained an independent predictor even after adjustments for traditional cardiac risk factors (age, sex, smoking, SBP, diabetes, HDL, and LDL) (adjusted model 1) (HR, 2.15; 95% CI: 1.56–2.95; *P* < 0.001) and eGFR with NTproBNP levels (adjusted model 2) (HR, 1.72; 95% CI: 1.07–2.78; *P* = 0.025) (Fig. 2f).

Elevated plasma levels of TMAVA and TML are both associated with incident cardiac death and transplantation risk independent of traditional risk factors (Figs. 1 and 2). Plasma TML demonstrated a significant correlation with TMAVA levels (Spearman’s correlation *r* = 0.40, *P* < 0.001) (Fig. 2g). Furthermore, to explore whether a high TML level is associated with a higher risk of future cardiovascular risk with a concomitant increase in TMAVA, we found that when TML was added to the adjustment model, elevated TMAVA (Q4) levels remained an independent predictor (HR, 1.75; 95% CI: 1.07–2.87; *P* = 0.026) (adjusted model 2, Fig. 2h).

The gut microbiota-derived metabolite TMAO is emerging as a potentially important cause of increased cardiovascular risk^25–27^. Kaplan–Meier analysis of TMAO levels presented a graded increased risk for a composite of cardiovascular death and heart transplantation (Supplementary Fig. 2a). Elevated TMAO levels were associated with significantly increased HR after adjusting for risk factors (age, sex, smoking, SBP, diabetes, HDL, LDL, eGFR, and NTproBNP) levels (Q4 vs. Q1: HR 1.78, 95% CI: 1.12–2.82) (Supplementary Fig. 2b), which is consistent with a previous study^26^. After adjustment for TMAVA, we found that TMAO was still associated with cardiovascular death (Supplementary Fig. 2b). Nonetheless, TMAVA still remained an independent predictor after adjusting for TMAO, indicating that TMAVA and TMAO may have different metabolic pathways and effects (Supplementary Fig. 2c). Next, the patients were stratified based on TMAVA and TMAO median (<median value vs. ≥median value). Kaplan–Meier survival plots showed that patients having the highest level of both TMAVA and TMAO experienced a significantly higher risk of HF, compared with those with the lowest level of both TMAVA and TMAO (Supplementary Fig. 2d).

### TMAVA aggravates cardiac hypertrophy and dysfunction induced by HFD and by transverse aortic constriction (TAC) on CD

**Fig. 3:**
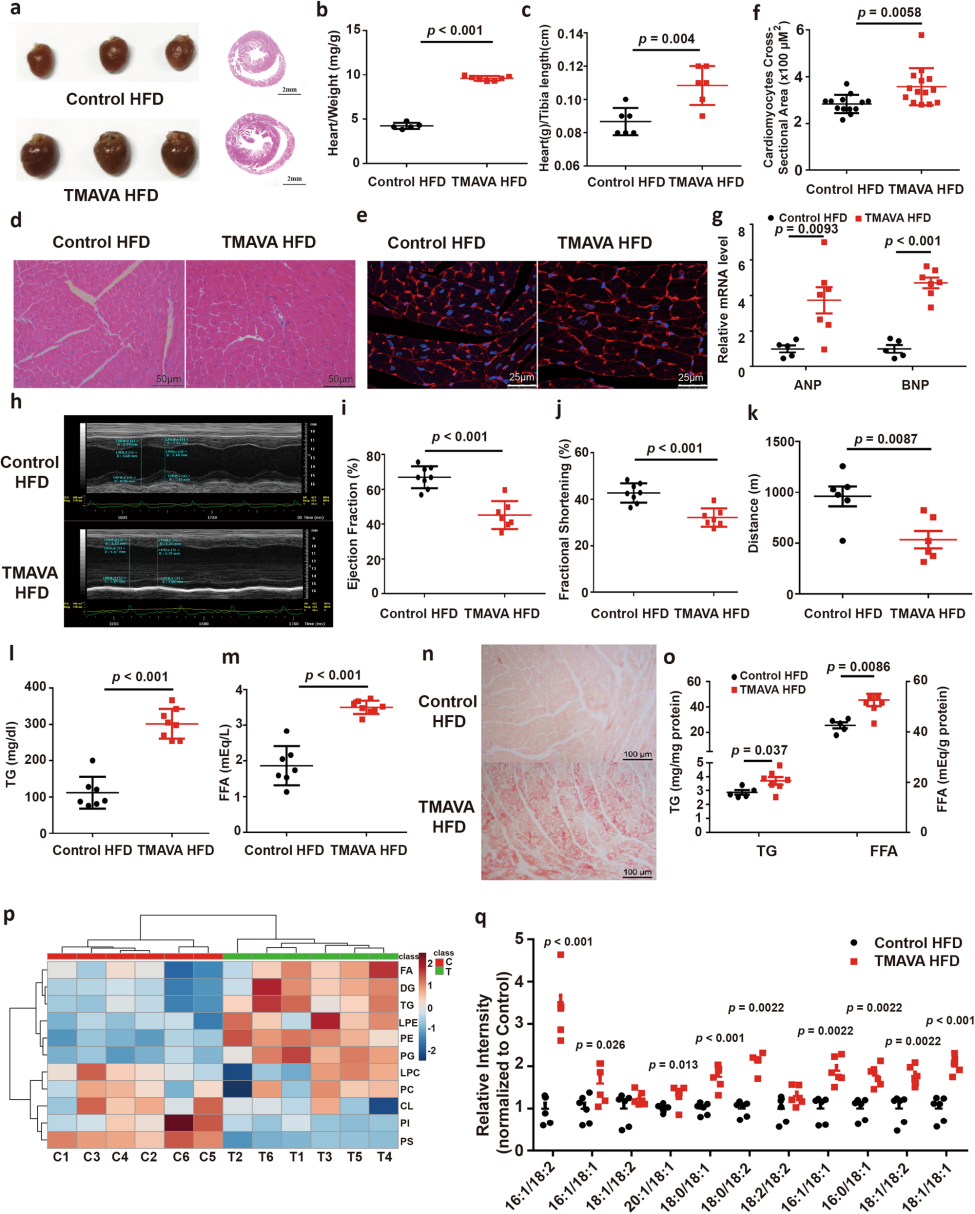
TMAVA aggravates cardiac hypertrophy and dysfunction in a high-fat diet. Mice were fed a HFD for 12 weeks, with the TMAVA group treated with 0.325% TMAVA (m/v %) in the drinking water in [**a**-**q**], *n* = 5–8/group. **a** Hearts from mice treated with TMAVA were enlarged (left) and the gross morphology of hearts in the TMAVA group showed increased ventricular dimensions and ventricular wall thickness in sections along the shorter axis of the heart (right). **b** The ratio of heart weight to body weight was significantly increased in the TMAVA-treated mice (*n* = 5 and 7). **c** The ratio of heart weight to tibia length was significantly increased in the TMAVA-treated mice (*n* = 6/group). **d**, **e** H&E (**d**) and Wheat germ agglutinin (WGA) staining (**e**) revealed myocardial cells were hypertrophic in the TMAVA-treated mice. **f** Quantitative analysis of cardiomyocyte cross-sectional area (CSA). Myocyte size was determined from CSA measurements of 100 myocytes per animal in five independent myocardial sections. **g** The mRNA levels of atrial natriuretic peptide (ANP) and brain natriuretic peptide (BNP) of TMAVA-treated mice hearts relative to controls (*n* = 5 and 7). **h** Representative echocardiographic images of the left ventricle showing ventricular dilation and cardiac dysfunction. **i**, **j** Ejection fraction (**i**) and fractional shortening (**j**) were assessed by echocardiography in control and TMAVA-treated mice (*n* = 8 and 7). **k** Distance covered during treadmill exercise to exhaustion in control and TMAVA treated mice (*n* = 6). **l**, **m** Plasma TG (**l**) and FFA (**m**) levels (*n* = 7 and 8). **n** Oil-red O staining revealed increased neutral triglyceride deposition in the heart from TMAVA-treated mice. **o** Myocardial TG and FFA contents were increased in TMAVA-treated mice (*n* = 5 and 7). **p** Comparative myocardial lipidomics was performed after 12 weeks of TMAVA treatment together with HFD (*n* = 6). Hierarchical cluster analysis heat map of lipid levels between control and TMAVA treated mice. Red indicates upregulation, and blue indicates downregulation. The columns and rows represent experimental heart samples and lipid species, respectively. Fatty acid (FA), diglyceride (DG), triacylglycerol (TG), lysophosphatidylethanolamine (LPE), phosphatidylethanolamine (PE), phosphatidylglycerol (PG), lysophosphatidylcholine (LPC), phosphatidylcholine (PC), cardiolipin (CL), phosphatidylinositol (PI), phosphatidylserine (PS). **q** DG species in control and TMAVA-treated mice. The experiments were performed as three replications. Statistical significance was evaluated by two-tailed unpaired Student’s *t* test [(**b**, **c**, **f**, **g**, **i**–**m**, **o**] or two-tailed nonparametric Mann–Whitney test [(**q**)]. (*P < 0.05, **P < 0.01, ***P < 0.001). Data are expressed as mean ± SEM. Source data are provided as a Source Data file.

To establish the specific contribution of the TMAVA metabolite to cardiac hypertrophy and HF associated with metabolic syndrome, mice were treated with or without TMAVA along with HFD for twelve weeks. After 12-weeks, the plasma TMAVA level was significantly elevated (9.52 μM vs. 113.5 μM). Heart weight was increased in the TMAVA-treated mice, compared with the untreated controls (Fig. 3a–c) along with a significant increase in the cross-sectional area of the cardiomyocytes (Fig. 3d–f). In addition, hypertrophic marker genes, including atrial natriuretic peptide and brain natriuretic peptide (BNP) were significantly upregulated (Fig. 3g). The echocardiographic assessment showed that left posterior wall thickness at diastole, LV dimension volume at systole, and LV mass were further increased in the TMAVA-treated HFD mice. EF and fractional shortening (FS) were further decreased in the TMAVA-treated mice (Fig. 3h–j). Accordingly, the TMAVA-treated mice showed markedly decreased exercise capacity (Fig. 3k). To address whether TMAVA could trigger cardiac hypertrophy in the absence of HFD, mice were also given 0.325% (m/v%) TMAVA in the drinking water while on a chow diet (CD). After 12 weeks, the heart weight was increased in the TMAVA-treated mice, compared with untreated controls (Supplementary Fig. 3a). The echocardiographic assessment showed that the left ventricular anterior wall (LVAW), posterior wall (LVPW) thickness, and LV mass were increased in the TMAVA-treated mice on CD. However, EF and FS remained unchanged indicating that cardiac function was still in a compensatory stage in the 12-week TMAVA-treated mice on CD (Supplementary Fig. 3b). Moreover, mice treated with TMAVA (m/v%) in the drinking water for 2 weeks, were subsequently subjected to transverse aorta constriction (TAC)-induced LV pressure overload. The heart weight was increased in the TMAVA-treated group (Supplementary Fig. 3c). The echocardiographic assessment showed that EF and FS were decreased in the TMAVA-treated mice (Supplementary Fig. 3d). These data suggest that TMAVA could drive cardiac hypertrophy in mice and that TMAVA treatment combined with HFD or TAC aggravates cardiac hypertrophy and promotes cardiac dysfunction.

### TMAVA treatment leads to myocardial lipid accumulation

After TMAVA treatment combined with HFD for 12 weeks, plasma triglycerides (TG) and FFA levels were increased significantly compared to the control group (Fig. 3l, m). Oil-Red O staining of frozen heart sections demonstrated significant levels of lipid droplets in the TMAVA-treated hearts but not in the control hearts (Fig. 3n). Consistent with this histological observation, the levels of the myocardial TG and FFA were 30% and 20% higher in the TMAVA-treated HFD samples than in the control HFD hearts (Fig. 3o). Furthermore, comparative myocardial lipidomics was performed after 12 weeks of TMAVA treatment combined with HFD. TMAVA-treated mice showed significantly altered lipid profiles compared with control, as indicated by principal component analysis (PCA) (Supplementary Fig. 4a). Hierarchical cluster analysis heat map indicated that TG and diglycerides (DG) were elevated (Fig. 3p and Supplementary Fig. 4b). FFA of medium- and long-chain length, which exert lipotoxicity, were also markedly increased in the heart (Supplementary Fig. 4c). DG has been implicated as the causative metabolites in lipotoxicity-induced insulin resistance through the action of protein kinase C^28^, and we found that DG species were increased in the TMAVA-treated mice (Fig. 3q).

### TMAVA inhibits carnitine metabolism and impairs myocardial energy homeostasis

**Fig. 4:**
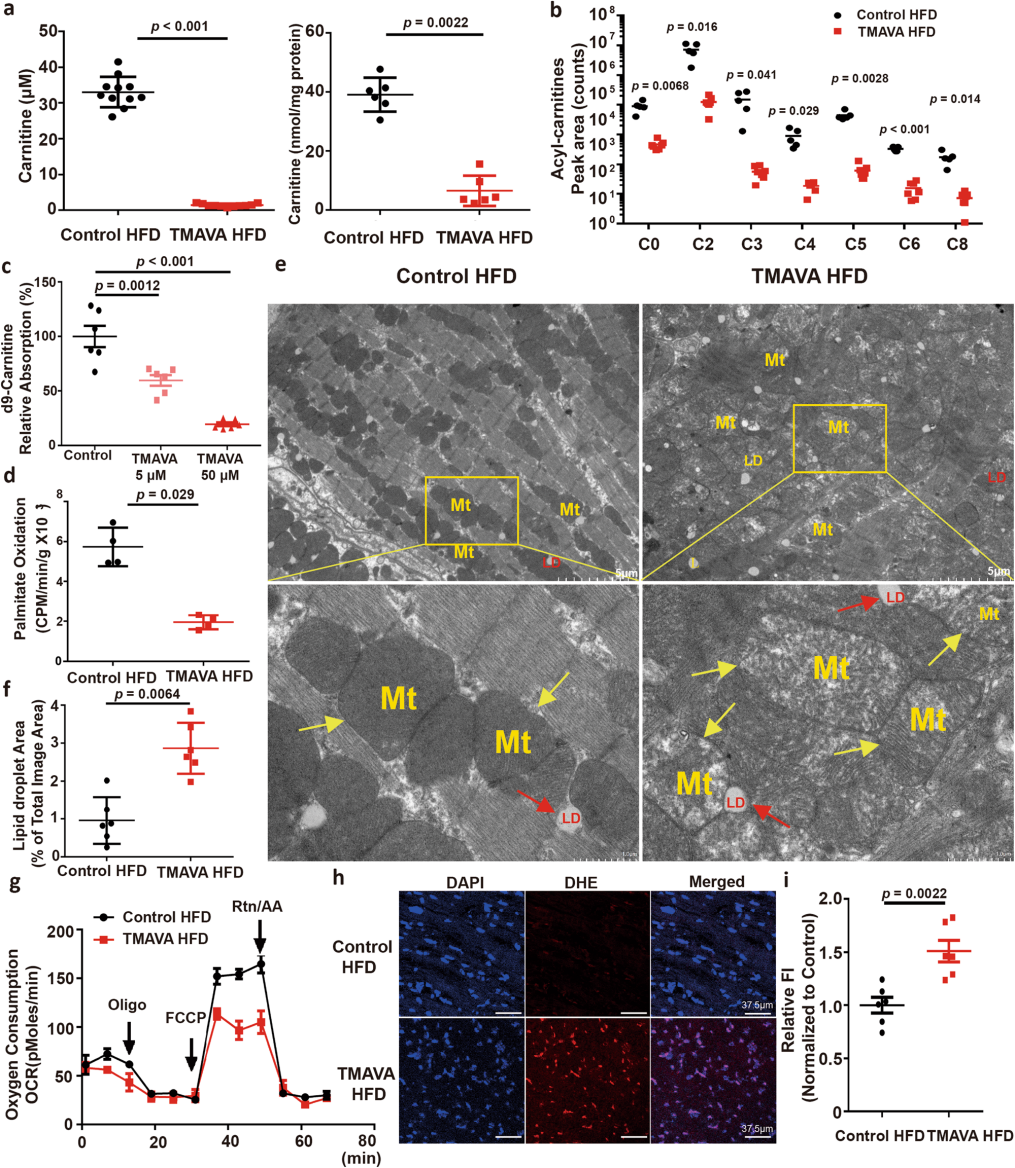
Fatty acid oxidation (FAO) was inhibited and myocardial mitochondria were dysfunctional upon TMAVA treatment. Mice were fed an HFD for 12 weeks, with the TMAVA group treated with 0.325% TMAVA (m/v%) in the drinking water in [(**a**, **b**) and (**d**)–(**i**)]. **a** TMAVA treatment decreased the carnitine content in plasma (left) (*n* = 11) and heart (right) (*n* = 6). **b** Acyl-carnitine levels were decreased in the heart. Cardiac individual acyl-carnitine species were measured by LC–MS (*n* = 5). **c** d9-carnitine uptake in primary neonatal rat ventricular myocytes (NRVM) was measured in the presence of 5 and 50 μM TMAVA (*n* = 6/concentration). **d** Cardiac fatty acid β-oxidation activity was measured ex vivo in control and TMAVA-treated mice using ^3^H-palmitic acid (*n* = 4). **e** Electron microscopy of hearts from control and TMAVA-treated mice fed an HFD for 12 weeks. LD lipid droplets, Mt mitochondria (*n* = 6). **f** Lipid droplet percentage (% of the total image area) was calculated with ImagJ (*n* = 6). **g** Respiratory rates of heart mitochondria isolated from WT mice and TMAVA-treated mice after 12 weeks on HFD. OCR was measured with palmitic acid as the substrate using Seahorse metabolic analyzer (*n* = 3). **h** Myocardial tissue ROS levels were detected using the oxidant sensitive probe dihydroethidium (DHE) (red). Nuclei were counterstained with DAPI (blue). **i** Quantification of the fluorescence intensity of DHE staining (Red) (*n* = 6/group). Statistical significance was evaluated by two-tailed unpaired Student’s *t* test [(**b**, **f**, **i**)], one-way ANOVA [(**c**)], or two-tailed nonparametric Mann–Whitney test [(**a**, **d**)]. (*P < 0.05, **P < 0.01, ***P < 0.001). Data are expressed as mean ± SEM. Source data are provided as a Source Data file.

TMAVA treatment significantly reduced plasma and cardiac carnitine (Fig. 4a). Consistent with the decrease in circulating carnitine, all acyl-carnitine species in the heart were significantly diminished, suggesting a depressed state of FAs β-oxidation (Fig. 4b). Carnitine is synthesized endogenously by hydroxylation of γ-butyrobetaine (γ-BB) through the γ-butyrobetaine hydroxylase (BBOX)^29^. Using surface plasmon resonance, we previously reported that TMAVA inhibited the binding of γ-BB to BBOX competitively, resulting in carnitine synthesis deficiency^21^. In the cross-sectional study involving HF patients (*n* = 1647, Supplemental Table 2), and controls (*n* = 1000), we found that TMAVA in HF patients displayed an increased trend (Supplementary Fig. 5a, left panel). Importantly, when the population was further divided into two groups, according to their γ-BB levels (“lower” or “higher”), TMAVA was elevated significantly in the patients with HF compared to the controls (Supplementary Fig. 5a, right panel) in those with higher γ-BB levels. Additionally, compared with the lowest quartile, the highest quartile of plasma TMAVA was associated with higher odds of HF (odds ratio [OR], 1.58; 95% CI: 1.12–2.23; *P* = 0.009). The multivariate-adjusted ORs (95% CIs) in model 1 (adjusted for age and sex) and model 2 (adjusted for model 1, smoking status, SBP, diabetes, HDL, LDL, and eGFR) were 1.67 (95% CI: 1.17–2.38) and 1.60 (95% CI: 1.00–2.56), respectively (Supplementary Table 8). Moreover, we found that plasma γ-BB showed a gradually increased mortality risk accompanied by graded γ-BB levels, particularly for the high quartiles (*P* < 0.0001) in a Kaplan–Meier analysis (Supplementary Fig. 5b). When compared with subjects in the lowest quartile of γ-BB levels, patients in the highest quartile (Q4) demonstrated a significantly increased risk of incident cardiac death and transplantation (HR, 2.61; 95% CI: 1.96–3.49; *P* < 0.001). Moreover, elevated γ-BB levels (Q4) remained an independent predictor even after adjustments for traditional cardiac risk factors (age, sex, smoking, SBP, diabetes, LDL, and HDL) (adjusted model 1) (HR, 2.66; 95% CI: 1.91–3.72; *P* < 0.001) and eGFR with NTproBNP levels (adjusted model 2) (HR, 1.78; 95% CI: 1.03–3.05; *P* = 0.037) (Supplementary Fig. 5c). In addition, patients were stratified based on TMAVA and γ-BB median (<median value vs. ≥median value). Kaplan–Meier survival plots showed that patients having the highest level of both TMAVA and γ-BB experienced a significantly higher risk of HF, compared with those with the lowest level of both TMAVA and γ-BB (Supplementary Fig. 5d). These results indicate that BBOX and γ-BB play an important role in HF.

Since carnitine is a hydrophilic quaternary amine, its passive permeability is very low, with carnitine/organic cation transporter 2 (OCTN2), a high-affinity transporter of carnitine, contributing to renal re-absorption and cardiac and hepatic carnitine uptake from circulation, respectively^30,31^. OCTN2 levels were highest in the kidney while the heart presented as the second tissue in abundance (Supplementary Fig. 6a). To determine whether TMAVA inhibits the carnitine uptake mediated by OCTN2, we established MDCK cells stably overexpressing human hOCTN2. The uptake of d9-carnitine in MDCK-hOCTN2 cells was evaluated in the presence of various concentrations of TMAVA. TMAVA reduced the uptake of d9-carnitine in a concentration-dependent manner with an IC50 of 4.15 μM (Supplementary Fig. 6b). TMAVA at 100 μM concentration has comparable suppressive effects as mildronate at 100 μM, a positive control to inhibit carnitine transport (Supplementary Fig. 6c). Additionally, we found that TMAVA also concentration-dependently inhibited the uptake of d9-carnitine in primary neonatal rat ventricular myocytes (NRVM), which confirmed its potential inhibitory effect on cardiac carnitine uptake^32,33^ (Fig. 4c). TMAVA level in human heart tissue was also measured and found comparable to the range found in a prior report^33^, with TMAVA concentrations of 33.8 µM, 7.99 μM, and 2.4 µM, in three random HF heart samples. These data indicated that TMAVA can inhibit carnitine metabolism through concurrent inhibition of BBOX (synthesis) and OCTN2 (uptake) in cardiac tissue, leading to myocardial lipid accumulation within the levels achieved pathophysiologically.

Proteomics was employed to discover changes in protein abundance and pathways enriched in regulated proteins in the heart upon TMAVA treatment. The TMAVA-treated mice on HFD showed significantly altered proteomic profiles compared with the HFD controls (Supplementary Fig. 7a). A total of 2638 heart proteins were examined, and the abundance of 457 of those proteins changed significantly. Biological process analysis of the cardiac proteome showed enrichment of proteins from oxidation-reduction and metabolic processes among the significantly regulated hits (Supplementary Fig. 7b and Supplementary Table 9). Enzymes involved in the TCA cycle were up-regulated (Supplementary Table 10). The enrichment analysis of GO cellular components also revealed that the proteins were mainly localized in the mitochondrion (Supplementary Table 11). Functional cluster analysis revealed that the significantly changed proteins were primarily related to energy metabolism (mitochondria and FAO) (Supplementary Table 12). In order to confirm altered FAO (Supplementary Fig. 7c), relative gene expression was measured. The expression of FAO genes, including carnitine palmitoyltransferase (CPT)1α, CPT1β, acyl-CoA dehydrogenase long-chain (ACADL), and acyl-CoA dehydrogenase very long chain (ACADVL) were increased in the heart tissue of the TMAVA-treated mice (Supplementary Fig. 7d). The TMAVA-treated mice showed suppressed lipogenic gene expression of FA synthase (FASN), likely in a compensatory fashion (Supplementary Fig. 7d). These data indicate that TMAVA treatment changed protein profiles in the FA metabolism in the mitochondria.

### FAO is impaired in TMAVA treated hearts

To investigate whether TMAVA treatment affects myocardial energy metabolism under the HFD condition, we assessed the rates of FAO in the two groups of mice. Mitochondrial FAO was decreased in the TMAVA-treated mice (Fig. 4d). In the treadmill exhaustion test, likely due to insufficient amounts of FFA being oxidized as an energy substrate for uncoupled mitochondrial respiration upon TMAVA treatment, TMAVA-treated mice had elevated FFA and FFA/glycerol molar ratio (Supplementary Fig. 7e, f). TMAVA-treated mice also displayed a significantly decreased glucose level after exercise performance, indicating that the use of glucose as an alternative energy substrate may be enhanced (Supplementary Fig. 7g). In addition, ultrastructural analysis of cardiac myocytes indicated that mitochondria were swollen with less electron-dense bodies in the TMAVA-treated mice (Fig. 4e) with increased accumulation of lipid droplets (Fig. 4f). We also examined respiratory rates in mitochondria isolated from control and TMAVA-treated mice. We found that heart mitochondria from TMAVA-treated mice exhibited defective respiratory chain with reduced maximal mitochondrial respiration (MMR) using palmitic acid as the substrate, indicating that mitochondrial respiration is severely impaired (Fig. 4g). The mitochondrial volume was also increased in the TMAVA-treated group (Supplementary Fig. 7h). Western blot results indicated that mitochondrial complexes (CI subunit NDUFB8, CII subunit SDH, CIII subunit UQCRC, and CV subunit ATP5A) showed a trend toward increased abundance after TMAVA treatment (Supplementary Fig. 7i, j). Myocardial tissue reactive oxygen species (ROS) levels were increased in the TMAVA-treated mice (Fig. 4h, i). Plasma oxidative stress marker 8-isoprostane was also increased in TMAVA-treated mice (Supplementary Fig. 7k). Thus, TMAVA treatment and subsequent lipid accumulation are associated with a reduction in FAO and increased oxidative stress in the mouse heart under HFD conditions.

### BBOX inactivation phenocopies TMAVA treatment

**Fig. 5:**
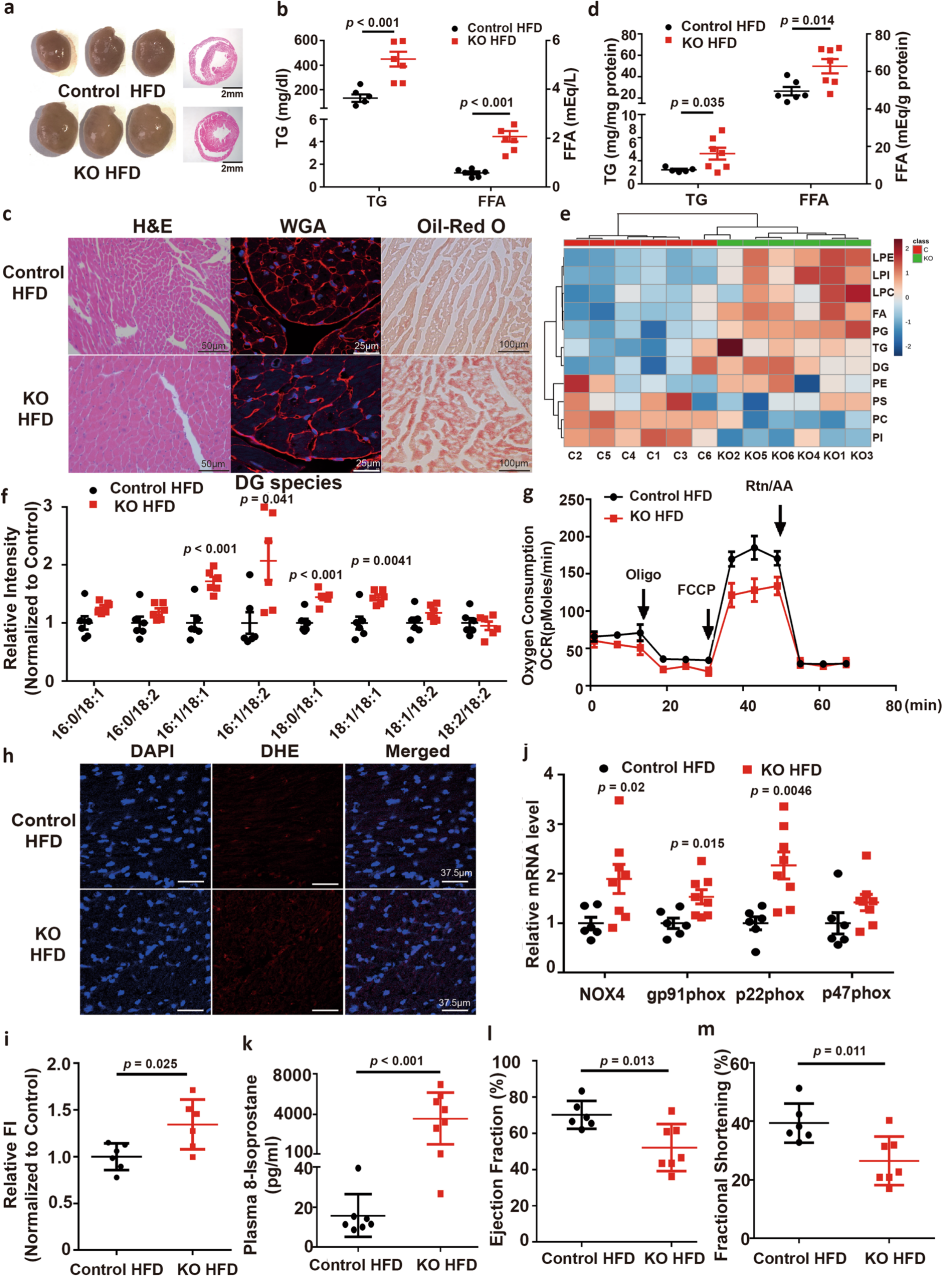
BBOX deficiency leads to cardiac hypertrophy, lipid accumulation, and oxidative stress. WT (control) and BBOX deficient (BBOX^−/−^) mice were fed an HFD for 12 weeks in [(**a**–**m**)]. **a** Gross morphology of the BBOX^−/−^ mice hearts shows enlargement compared with controls. **b** Plasma TG and FFA levels were measured after 16 h fasting (*n* = 6 and 7). **c** H&E (left), WGA (middle), and Oil-red O (right) staining revealed hypertrophic myocardial cells and increased neutral triglyceride deposition in the heart of BBOX^−/−^ mice. **d** Myocardial TG and FFA contents were increased in the BBOX^−/−^ mice (*n* = 6 and 7). **e** Hierarchical cluster analysis heat map of differential levels for the indicated lipids between control and BBOX^−/−^ mice (*n* = 6). Red indicates upregulation and blue indicates downregulation. The columns and rows represent experimental heart samples and lipid species, respectively. **f** DG species in control and BBOX^−/−^ mice (*n* = 6). **g** Respiratory rates of heart mitochondria isolated from WT mice and BBOX^−/−^ mice after 12 weeks’ treatment with HFD. OCR was measured with palmitic acid as the substrate using Seahorse metabolic analyzer (*n* = 3). **h** Myocardial tissue ROS levels were detected using the oxidant sensitive probe dihydroethidium (DHE) (red) and DAPI (blue) counterstained the nuclei. **i** Quantification of the fluorescence intensity of DHE (Red) (n = 6/group). **j** RT-qPCR of mRNA levels of cardiac genes involved in oxidation (Nox4, gp91phox, p22phox, and p47phox) (*n* = 6 and 8). **k** The plasma oxidative stress marker 8-Isoprostane was measured after 12 weeks on HFD (*n* = 7 and 8). **l**, **m** Ejection fraction (**l**) and fractional shortening (**m**) were assessed by echocardiography in control and BBOX^−/−^ mice after 12 weeks on HFD (*n* = 6 and 7). Statistical significance was evaluated by two-tailed unpaired Student’s *t* test [(**b**, **j**, **i**, **l**–**m**)] or two-tailed nonparametric Mann–Whitney test [(**d**, **f**, **k**)]. (*P < 0.05, **P < 0.01, ***P < 0.001). Data are expressed as mean ± SEM. Source data are provided as a Source Data file.

To provide genetic evidence that BBOX is the molecular target for TMAVA to induce FAO impairment, BBOX-deficient mice were generated by CRISPR–CAS9 mediated deletion of exon 2 in the mouse BBOX gene^21^ (Supplementary Fig. 8a). We found that the heart morphology of the BBOX knockout mice was increased compared with control mice in HFD for 12 weeks (Fig. 5a). Serum TG and FFA contents were elevated, in a fashion comparable to TMAVA treatment (Fig. 5b vs. Fig. 3l, m). H&E, WGA, and Oil-red O staining revealed hypertrophic myocardial cells and increased neutral lipid deposition in the BBOX^−/−^ mice heart (Fig. 5c), which is consistent with and recapitulates the TMAVA treatment (Fig. 3). In agreement with the histological observations, the level of the myocardial TG and FFA were 40% and 30% higher in the BBOX^−/−^ mice on HFD than in the WT mice on HFD (Fig. 5d). Moreover, comparative myocardial lipidomics was performed in the two groups after 12 weeks on HFD. BBOX^−/−^ mice showed significantly altered lipid profiles compared with WT controls, as established from PCA and heat map analysis (Fig. 5e and Supplementary Fig. 8b). We found that TG, DG, FA, lysophosphatidylethanolamine (LPE), lysophosphatidylcholine (LPC), lysophosphatidylinositol (LPI) were increased, while phosphatidylcholine (PC), and phosphatidylinositol (PI) were decreased in the BBOX knockout hearts compared with the WT controls (Supplementary Fig. 8c). FFA of medium- and long-chain length were also markedly increased in the heart (Supplementary Fig. 8d). DG species, which exert cardiac lipotoxicity, were also increased in the BBOX^−/−^ mice (Fig. 5f).

Ultrastructural electron microscopy analysis of cardiac myocytes indicated that mitochondria were swollen in the BBOX-deficient mice (Supplementary Fig. 9a) with increased lipid droplets accumulation (Supplementary Fig. 9b). We also found that mitochondria isolated from BBOX^−/−^ mice exhibited defective respiratory chain with reduced MMR using palmitic acid as the substrate, indicating that mitochondrial respiration is severely impaired in these mice (Fig. 5g). The mitochondrial volume was also increased in the BBOX^−/−^ group (Supplementary Fig. 9c). In agreement with the increased mitochondrial volume, the relative amount of mitochondrial complexes (CI subunit NDUFB8, CII subunit SDH, CIII subunit UQCRC, and CV subunit ATP5A) showed a trend towards increased abundance in the BBOX^−/−^ mice (Supplementary Fig. 9d, e). The amounts of mtDNA content were increased, as measured by quantitative PCR of mtDNA-encoded gene mt-cytb (encoding cytochrome b) (Supplementary Fig. 9f). Accordingly, myocardial tissue ROS levels were increased in BBOX^−/−^ mice (Fig. 5h, i), and mRNA levels of cardiac genes involved in oxidative stress (NOX4, gp91phox, p22phox, and p47phox) were also elevated (Fig. 5j). The plasma oxidative stress marker 8-isoprostane was also increased in the BBOX^−/−^ mice (Fig. 5k). EF and FS were further decreased in the knockout mice (Fig. 5l, m). Taken together, these results indicate that BBOX^−/−^ mice recapitulated the phenotype of mice treated with TMAVA, which indicates that TMAVA caused cardiac lipid accumulation and hypertrophy through BBOX.

### Exogenous carnitine supplementation reverses TMAVA-induced cardiac hypertrophy

**Fig. 6:**
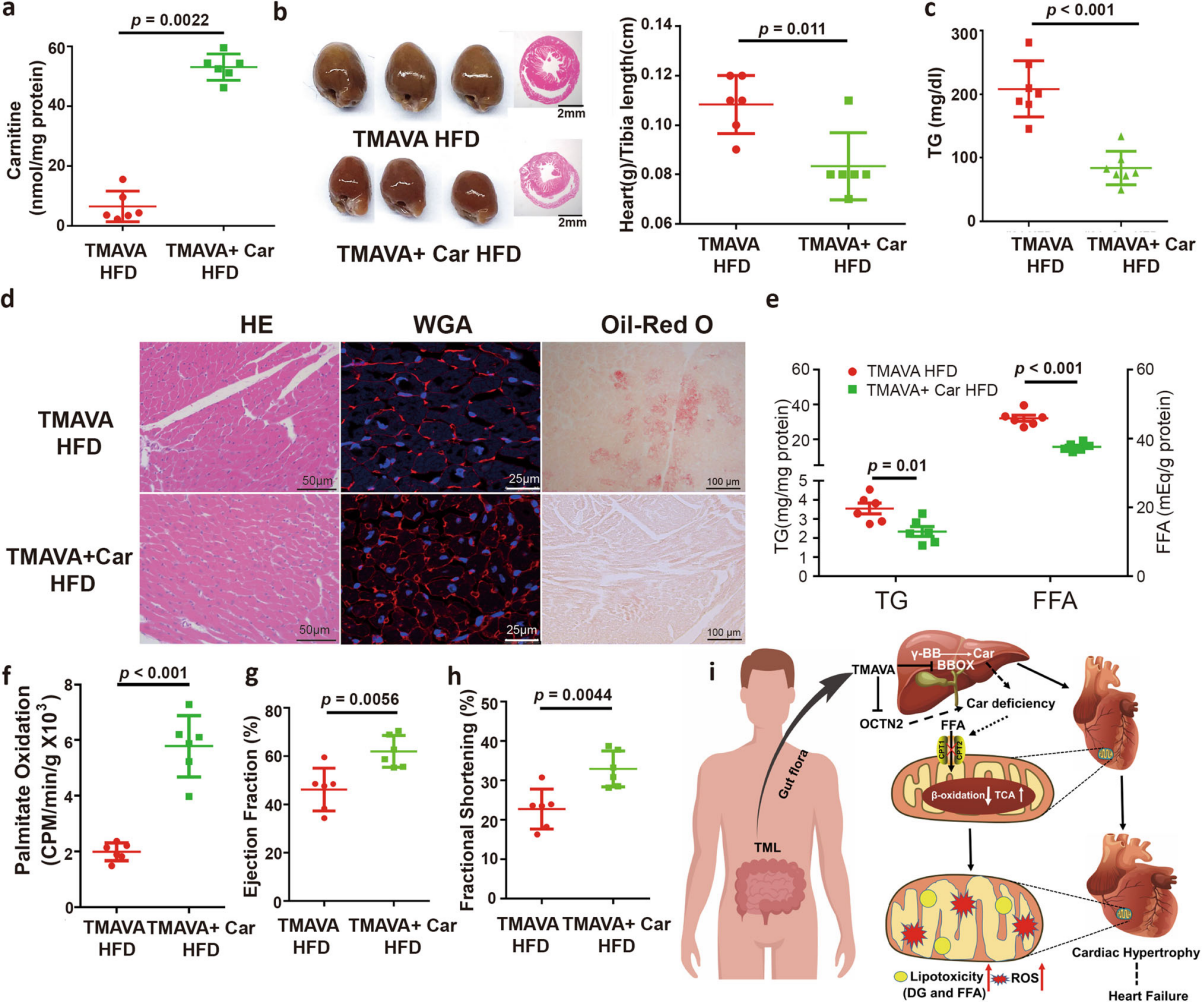
Exogenous carnitine supplementation reverses TMAVA induced cardiac hypertrophy. TMAVA-treated mice [0.325% (m/v %) in the drinking water]. Mice were fed an HFD with or without 0.325% carnitine (m/v %) in the drinking water for 12 weeks in [(**a**–**h**)]. **a** Carnitine level in heart tissue was measured after 12 weeks of treatment (*n* = 6). **b** The hearts from the carnitine-treated group (TMAVA + Car) group were smaller (left) and the ratio of heart weight to tibia length (right) was significantly decreased in that group (*n* = 6/group). **c** Plasma TG was decreased in the carnitine-supplemented mice (*n* = 7). **d** H&E (left), WGA (middle), and Oil-red O (right) staining revealed myocardial cells with reduced hypertrophy and decreased neutral triglyceride deposition in the hearts of mice treated with TMAVA + Car. **e** Myocardial TG and FFA contents were decreased in the group with carnitine treatment (*n* = 6). **f** Cardiac fatty acid β-oxidation activity was measured ex vivo in control and TMAVA-treated and TMAVA + Car-treated mice using ^3^H-palmitic acid (*n* = 6). **g**, **h** Ejection fraction (**g**) and fractional shortening (**h**) were assessed by echocardiography in the TMAVA and TMAVA + Car mice after 12 weeks (*n* = 6). **i** Summary scheme outlining the proposed pathway by which TMAVA promotes cardiac hypertrophy. TMAVA is metabolized from TML by the microbiota. TMAVA competes with γ-BB for binding to the BBOX and suppresses endogenous carnitine synthesis from γ-BB in the liver and uptake by OCTN2 in the kidney and heart. Decreased carnitine levels inhibit FFA oxidation, leading to myocardial lipid accumulation. Subsequently, lipotoxicity results in excess oxidative stress and mitochondrial dysfunction. With FFA oxidation inhibited, there is likely a compensatory increase in glucose metabolism. The pathologic metabolism results in cardiac hypertrophy and eventual progression to HF. Statistical significance was evaluated by two-tailed unpaired Student’s *t* test [(**c**, **e**, **f**, **g**–**h**)] or two-tailed nonparametric Mann–Whitney test [(**a**, **b**)]. (*P < 0.05, **P < 0.01, ***P < 0.001). Data are expressed as mean ± SEM. Source data are provided as a Source Data file.

To examine whether carnitine supplementation can reverse TMAVA-induced cardiac hypertrophy, we treated WT mice on HFD with TMAVA and with or without carnitine in their drinking water. Carnitine supplementation significantly reversed the TMAVA-induced decrease in carnitine in the heart (Fig. 6a). The pathological effects of TMAVA in cardiac hypertrophy and lipid contents were significantly abolished by carnitine supplementation, as evidenced by heart weight/tibia length and TG level (Fig. 6b, c), which become comparable to those in control mice without TMAVA treatment (Fig. 3c vs Fig. 6b). H&E, WGA, and Oil-red O staining revealed abolished hypertrophy of myocardial cells and decreased neutral lipid deposition in the carnitine-treated mice (Fig. 6d). The level of the myocardial TG and FFA in the carnitine-supplemented group was also reduced by 20%, to levels comparable to control mice without TMAVA treatment in HFD (Fig. 3o vs. Fig. 6e). Mitochondrial FAO was also increased in the carnitine-treated mice (Fig. 6f). Consistent with the histological observations, the echocardiographic assessment showed that EF and FS were also reversed in the carnitine group compared with the TMAVA-only group (Fig. 6g, h), although the carnitine group displayed a lower EF and FS compared with the group in HFD alone (Fig. 3 i, j vs. Fig. 6g, h). Therefore, exogenous carnitine supplementation can reverse TMAVA-induced cardiac hypertrophy.

## Discussion

The present study demonstrates that TMAVA, a microbially produced metabolite, accelerates cardiac hypertrophy. The results indicate the following: (1) Higher TMAVA level is accompanied with gradually increased cardiac mortality risk in our HF cohort; (2) *E. faecalis* level was increased in HF patients; (3) Mice treated with TMAVA show aggravated cardiac hypertrophy and dysfunction on HFD; (4) TMAVA inhibits carnitine synthesis and uptake, leading to myocardial FAO inhibition and lipid accumulation; (5) TMAVA treatment leads to cardiac mitochondrial dysfunction and accumulation; (6) BBOX deficiency inhibits FAO and leads to myocardial lipid accumulation, which indicates that TMAVA functions through BBOX; and (7) Exogenous carnitine supplementation reverses TMAVA-induced cardiac hypertrophy. Taken together, these results demonstrate that the gut microbiota-derived TMAVA is a key determinant in the development of cardiac hypertrophy through inhibition of carnitine synthesis and subsequent FFA oxidation. TMAVA may be a therapeutic target for secondary prevention in HF patients (Fig. 6i).

HF is associated with a massive increase in levels of pathogenic bacteria and fungi in the gut, as well as increases in intestinal permeability and inflammation. Previous studies have found that colonies of pathogenic bacteria in the gut show a correlation with the severity of HF. Compared with healthy individuals, patients with HF had more colonies of pathogenic bacteria^34,35^. Moreover, patients with HF had an intestinal overgrowth of pathogenic bacteria, which causes chronic intestinal wall and systemic inflammation leading to increased intestinal permeability^34,36,37^. On the other hand, specialized metabolites derived from the gut microbiota act in concert with the host metabolism. Previous studies found that TMAO, a microbial metabolite, contributes to risk prediction of in-hospital mortality in acute HF when combined with current clinical risk scores that include an adjustment for renal function^26^. Those data provided evidence for the importance of the gut microbiota in the pathogenesis of heart disease and suggested a gut-heart axis. In our study, we found that TMAVA was generated from TML by the gut microbiota (Fig. 1f–h). More importantly, as compared with subjects in the lowest quartile of TMAVA, patients in the highest quartile demonstrated a significantly worse prognosis for HF. Following adjustments for traditional cardiovascular disease (CVD) risk factors, NTproBNP and eGFR, elevated plasma levels of TMAVA remained a significant independent predictor for risk of HF (Fig. 1a, b). Additionally, we found that eGFR is negatively correlated with quartiles of TMAVA in plasma, which indicates TMAVA may be a readout of renal function which, in turn, is known to be an independent risk factor for HF^38^. However, TMAVA still remained significant after eGFR adjustment, indicating that elevated plasma TMAVA level is associated with incident cardiac death and transplantation risk, independent of renal function, in HF patients. Since TMAVA is also a low molecular weight compound that may be easily filtered by the kidney, elevated TMAVA levels may be due to diminished renal function and we also observed that eGFR is negatively correlated with quartiles of TMAVA in plasma. Several previous studies have suggested a shift in the gut microbiota in chronic kidney disease^39,40^, as well as altered intestinal barrier function in this setting^41^. Thus, it is plausible that an increased intestinal metabolism and absorption of TMAVA could contribute to the elevated TMAVA concentrations in patients with impaired renal function^42^. In the future, additional basic studies intended to investigate the effects of TMAVA in renal function and large-scale cross-sectional studies specifically designed to examine the predictive value of TMAVA for cardiovascular outcomes in patients with kidney disease should be developed.

Recently, Li and his colleagues discovered a plasma metabolite, TML, associated with CVD risks, using an untargeted metabolomics approach^22^. TML levels were associated with incident (3-year) major adverse cardiovascular event risks and incident (5-year) mortality risk independent of traditional cardiovascular risk factors^22^. TML is a relatively abundant posttranslational modification of proteins in both animals and plants alike; it is found in a wide array of animal and vegetable matter and can serve as a nutrient precursor for gut microbiota-dependent generation of trimethylamine (TMA) and its subsequent conversion to TMAO in the liver in vivo^22^. In our study, we found that TML levels were associated with a gradually increased mortality risk at higher levels, which is consistent with previous studies^22,23^. However, Li et al.^22^ showed that fecal cultures from mice and humans (omnivores and vegans) promote the conversion of TML to TMA, albeit with a low transformation potential (TML « choline). Furthermore, unlike chronic dietary choline intake, TML supplementation in mice failed to elevate plasma TMAO^22^. In this study, we found that TMAVA displayed markedly (50- to 100-fold) higher peak levels than TMAO when metabolized from TML in vivo (Fig. 1g). These results directly demonstrate that oral TML can preferentially serve as a precursor for TMAVA from the microbiota rather than liver TMAO in vivo. More importantly, we found that when TML was added to the adjustment model, elevated TMAVA (Q4) levels remained an independent predictor (Fig. 2h). These results indicate that TMAVA, rather than TMAO, may be the main metabolite derived from TML and may play an important role in CVD and HF.

Impaired FAO will result in excess accumulation of lipids, termed lipotoxic cardiomyopathy, leading to heart dysfunction. It is argued that patients with type 2 diabetes, metabolic syndrome, and obesity accumulate excess intramyocardial lipids and exhibit decreased systolic or diastolic function^43^. In our study, mitochondrial FAO is impaired in the TMAVA-treated hearts (Fig. 4d), likely from the combined effect of concurrent TMAVA-dependent inhibition of BBOX^21^, leading to reduced intracellular carnitine synthesis, and of OCTN2, resulting in impaired uptake (Supplementary Fig. 6)^32,33^. Additionally, we found that TMAVA could drive cardiac hypertrophy in mice in CD, although the cardiac function was in a compensatory stage. Consistently, TMAVA exacerbated cardiac dysfunction when administered along with either TAC (pressure overload, in CD) or HFD (metabolic dysfunction). This is reminiscent of a two-hit effect, as proposed in the model of non-alcoholic fatty liver disease^44^ and HF with preserved EF^45^.

TMAVA-dependent decrease in FAO leads to myocardial lipid accumulation (Fig. 3n, o). Moreover, intramyocardial lipid accumulation is associated with contractile dysfunction in heart tissues from patients with nonischemic HF^46^. Mass spectrometry assessment of lipidomics revealed that DG content in the hearts of TMAVA-treated mice and BBOX^−/−^ mice were substantially elevated compared with the corresponding control hearts (Fig. 3q and Fig. 5f). A previous study reported that DG has been implicated as the causative metabolites in lipotoxicity-induced insulin resistance through the action of protein kinase C (PKC)^28^. PKC downstream signaling has been implicated in apoptosis, insulin resistance, autophagy, and the development of pathological cardiac hypertrophy^47^. GPCR agonists increase the production of DG, resulting in sustained PKC activation in the cardiomyocyte^48^. Excess DG also activates nicotinamide adenine dinucleotide phosphate (NADPH) oxidase (NOX2)^49^. NOX2-derived ROS may promote FFA-induced dysfunction of pancreatic beta-cells through the JNK pathway^50^. In line with these findings, NADPH oxidase activity was significantly higher, resulting in enhanced superoxide generation, as assessed by DHE staining^51^. Enhanced mitochondrial ROS generation reduced mitochondrial respiration and ATP synthesis. It is well-known that mitochondria are the major ROS generating sources. In our study, we found that myocardial tissue ROS levels were increased in TMAVA-treated and BBOX^−/−^ mice and mRNA levels of oxidative cardiac genes (Nox4, gp91phox, p22phox, and p47phox) were also elevated, indicating excess oxidative stress (Fig. 4h and Fig. 5h–j). Lipotoxicity also provokes the permeabilization of the outer mitochondrial membrane^52^, leading to the cessation of the bioenergetic and redox functions of mitochondria^53^. Lipid overload also increased AKAP121 ubiquitination, modulated DRP1 phosphorylation, and altered OPA1 processing. ROMO1 (ROS modulator 1), a mitochondrial protein, is also a redox-sensitive factor that regulates mitochondrial morphology by affecting OPA1 cleavage and oligomerization^54^. In fact, in the TMAVA-treated and BBOX^−/−^ mice, the mitochondria respiration is severely impaired (Fig. 4g and Fig. 5g). These results indicate that TMAVA treatment causes oxidative stress and alterations in mitochondrial structure and function.

The rate-limiting step in endogenous carnitine biosynthesis involves the hydroxylation of γ-BB by the BBOX to yield carnitine both in eukaryotes and in prokaryotes^29,55^. On the other hand, carnitine can be metabolized to γ-BB, but only by the gut microbiota, and γ-BB can be further cleaved to produce TMA. These are two different metabolic pathways. The former contributes to energy metabolism and shows health benefits and the latter contributes to TMAO biosynthesis in the gut, which shows pro-inflammatory effects leading to disease pathogenesis, including atherosclerotic CVD, metabolic syndrome, etc. In humans, the balance between the two pathways affects health outcomes. In our study, we found TMAVA competes with γ-BB for binding to the BBOX, thus inhibiting carnitine synthesis (Fig. 4a, b). In addition, TMAVA can efficiently inhibit the uptake of carnitine in cardiomyocytes via OCTN2 (Fig. 4c). A recent study also found that cardiac overexpression of OCTN2 using an adeno-associated viral vector significantly improved EF and reduced interstitial fibrosis in mice subjected to TAC, underscoring the important role of OCTN2 in cardiac metabolism^56^. With the ensuing carnitine deficiency, circulating acyl-carnitine species also decreased significantly, indicating inhibited FAO. We found that HFD aggravated TMAVA-induced cardiac dysfunction, which, in the context of our overall findings, can be mainly attributable to HFD increasing FA sources in the heart combined with the fact that FAO was inhibited due to reduced carnitine metabolism brought about by the TMAVA-treatment. FAO is a major energy source for the adult mammalian heart^57^. Decreased FAO with increased reliance on glucose is a hallmark of metabolic remodeling that occurs in pathological cardiac hypertrophy and is associated with decreased myocardial energetics and impaired cardiac function^58,59^. Carnitine plays an indispensable role in FAO through facilitating the transport of the acyl-carnitine molecules catalyzed by CPT^9^. Patients with systemic carnitine deficiency have severe cardiomyopathy associated with reduced levels of carnitine while oral carnitine supplementation can dramatically reduce the cardiac size and relieve cardiomyopathy^60–62^. Carnitine is also an antioxidant, acting as a free radical scavenger to protect cells from ROS^63^. Treatment with carnitine has been shown to protect against ischemia-reperfusion injury and improve exercise tolerance and activity levels in patients with CVD^63,64^.

In this study, we found that exogenous carnitine supplementation reverses TMAVA-induced cardiac hypertrophy, indicating carnitine availability is vital in cardiac metabolism. However, the evidence supporting the cardioprotective efficacy of carnitine is controversial^65,66^. Recent studies found that the metabolism of dietary carnitine by the intestinal microbiota, leading to TML and TMA, thus promoting TMAO generation, enhances atherosclerosis^27^. Additionally, the gut microbiota seems to play a crucial role in accounting for the distinct and adverse consequences between dietary carnitine and CVD by promoting the generation of TMAO. In agreement, plasma carnitine levels predicted increased risks for incident major adverse cardiac events, but only in individuals whose TMAO levels were also elevated^27^. Omnivorous individuals were found to produce substantially more TMAO than vegans or vegetarians when challenged with comparable amounts of deuterium-labeled carnitine, and clinical studies comparing individuals before and following treatment with an oral cocktail of antibiotics showed that intact gut microbiota plays an obligatory role in dietary carnitine conversion into TMAO in humans^27^. Intriguingly, proportions of microbial taxa belonging to the *Clostridiaceae* and *Peptostreptococcaceae* families within feces were observed to be positively associated with both omnivorous dietary patterns and blood levels of TMAO in humans, making it tempting to speculate that these taxa may be involved in the initial metabolism of carnitine to TMA in the gut^27,67^. Distinct genetic backgrounds of mice and facility environments show differences in microbiota^68^ achieving a degree of diversity comparable to that observed in human populations, even for genetically inbred lines. Future studies to systematically address these microbial-driven host phenotypes should rely on variations of emerging technologies for normalization and standardization of microbiome studies^68^ combined with pharmacokinetic studies. They should be set up to address the individual contribution of given bacterial communities to the effects of carnitine supplementation via different routes on the balance between TMAO and TMAVA and the ensuing implications to cardiometabolic diseases. In spite of those caveats, and of relevance for clinical applications, a recent study^69^ also found that compared with the oral route, parenteral administration of carnitine has no obvious effects on TMAO levels, and no adverse impact on atherosclerotic lesion progression in HFD-fed ApoE^−/−^ mice. Subcutaneous administration of carnitine avoided microbial metabolism and was safe in terms of atherosclerosis. In addition, the bioavailability of carnitine by parenteral administration is higher than that of oral administration (100% vs. 5–18%). Therefore, although carnitine is essential for cardiac metabolism, it is possible that diverse gut microbiota composition may enhance the generation of proatherogenic TMAO from carnitine and promote CVD risk. This may have important clinical implications. Among patients at high risk for CVD, for example, end-stage renal disease subjects undergoing maintenance hemodialysis, routine carnitine supplementation is mandatory. For these patients, parenteral administration, rather than oral administration, maybe the preferred option^69^. Similarly, it is possible that parenteral administration of carnitine could have substantial effects in preventing TMAVA-associated cardiac remodeling and HF in heart disease patients.

In summary, it is remarkable that numerous large-scale epidemiological studies have noted a relationship between gut microbiota, lipotoxicity, and an enhanced risk for HF. The present work reveals the clinically relevant discovery that the gut microbiota, through the generation of TMAVA, potentially participate in modulating cardiac hypertrophy and the eventual progression to HF. These results thus suggest potential therapeutic targets and the refinement of nutritional interventions for cardiac hypertrophy. Specifically, the present study suggests that targeting the gut microbial TMAVA pathway as a treatment strategy has the potential to temper cardiac hypertrophy progression associated with elevated TMAVA, be it via dietary manipulation, alteration of the microbial community with a probiotic or prebiotic, or direct pharmacological inhibition of microbial enzymes involved in TMAVA production. Studies addressing this possibility represent an attractive future area of investigation. Therefore, the discovery of a link connecting TMAVA generation, gut microbiota metabolism and HF risk could have broad health-related implications.

## Methods

### Research subjects

The learning cohort leading to the discovery of TMAVA was described in our recent publication^21^. TMAVA levels in patients with hypertension in that cohort were analyzed in this study. The identification of hypertensive patients was based on a clearly documented medical history of hypertension with a systolic blood pressure ≥ 140 mm Hg or a diastolic blood pressure ≥ 90 mm Hg^70^. The baseline characteristics of subjects in the learning cohort are presented in Supplementary Table 1. The study was designed and carried out in accordance with the principles of the declaration of Helsinki and approved by the Ethics Committee of Beijing TianTan Hospital. All patients provided written informed consent.

The validation cohort, on the other hand, is a prospective population-based cohort study. At recruitment, 1647 participants with HF were enrolled between 2008 and 2017, corresponding to the longest 84-month follow-up visit (Supplementary Table 2). Follow-up for mortality and specific outcome of adverse events were implemented by trained interviewers. The participants were tracked until the first occurrence of defined outcomes, including cardiac death and heart transplantation. Inclusion and diagnosis of these patients were assessed by cardiologists from the Tongji Hospital Affiliated to Tongji Medical College, Huazhong University of Science and Technology, Wuhan, China. Indications for the enrollment of HF and exclusion criteria have been reported previously^71^. Briefly, subjects with clinically significant valvar heart disease, acute myocardial infarction, or unstable angina within one month, as well as those with malignant tumors, were excluded from the study. Patients with severe coronary heart disease without complete revascularization therapy were not specifically excluded. Concomitantly, control subjects without significant cardiac disease consecutively screened with coronary angiogram and echocardiography were selected at random in the same hospital from October 2013 to March 2017. All blood samples were collected at the fasting state and stored at −80 °C immediately until analysis. The study was designed and carried out in accordance with the principles of the declaration of Helsinki and approved by the Ethics Committee of Tongji Medical College. All subjects provided informed consent.

### Experimental animals

Starting at 6 weeks of age, wild-type male C57BL/6J mice were fed an HFD (45% of calories from fat; Cat: D12451, Research Diets, Inc.) or CD (10% of calories from fat; Cat: D12450H, Research Diets, Inc.) for 8 weeks along with the indicated interventions. The sample sizes for all animal studies are indicated in each figure legend. Mice were housed in a climate-controlled environment with a 12-h light/dark cycle and free access to food and water. Germ-free mice were purchased and maintained in the Institute of Laboratory Animal Science, Chinese Academy of Medical Sciences (CAMS) & Peking Union Medical College (PUMC)^72^. BBOX^−/−^ were generated through CRISPR-Cas9 genome editing^21^. After 12 weeks of HFD feeding, blood samples were drawn from mice and commercially available assay kits were used for the determination of glucose (Cat:240, BioSino Inc., China), FFA (Cat: 294–63601, Wako Chemicals Inc., Japan), TC (Cat: 180, BioSino Inc., China) and TG (Cat: 220, BioSino Inc., China) levels using commercially available assay kits^11^. For histology, hearts were sectioned at a thickness of 7 µm on a cryostat for Oil-Red O staining. The paraffin-embedded heart was sectioned at a thickness of 5 µm and stained with hematoxylin and eosin (H&E). The data were evaluated blindly by two independent investigators. All protocols for mouse experiments were in compliance with all relevant ethical regulations and were approved by the Ethics Committee of Animal Research, Peking University Health Science Center (LA2011-061 and LA2017004).

### Untargeted metabolomics

The procedures were published^21^ and are summarized here. Briefly, for plasma extraction 100 μL plasma was aliquoted to a 1.5-ml Eppendorf tube. Totally, 400 μL methanol (pre-chilled to −80 °C) to the supernatant and gently shaken to mix and incubate at −80 °C for 1–2 h. Next, the sample was centrifuged at 14,000*g* for 10 min (4–8 °C), the supernatant was transferred to a new Eppendorf tube and dried under-speed vac without heating. The dried samples were kept frozen at −80 °C freezer until use within 1-week. For metabolomics analysis: The dried samples were re-dissolved with 50 μL 20% methanol in water and centrifuged at 14,000*g* for 10 min at 4 °C. Then 40 μL of the supernatant was transferred to an MS vial with a plastic insert. Untargeted metabolites screening was performed on LC-Q Exactive Orbitrap MS (Thermo Fisher, USA) as the previous description^21^. For data processing: Metabolites were identified and quantified based on an in-house database using Tracefinder v3.1 (Thermo Fisher Scientific, USA). For polar metabolite analysis, two levels of identification were performed simultaneously using Tracefinder. Metabolites were first potentially assigned according to the endogenous MS database by accurate masses. At the same time, those that can match with the spectra in the fragment database were confirmed at MS/MS level. Ten ppm and 15 ppm mass tolerance were applied for precursor and fragment matching, respectively. Still, a tolerance of 0.25 min retention time shift was allowed for quantitation. The instrument stability was monitored using QC samples.

### Lipidomics

Lipids were extracted according to a modified method of Bligh and Dyer^73,74^. Briefly, 50 mg heart tissue samples were homogenized in 500 μL cold phosphate buffer saline (PBS). Then lipids were extracted by adding chloroform:methanol (2:1, v/v). Glass tubes (5 mL) were used here to avoid polymer contamination. Samples were vortexed for 2 min, followed by 5 min centrifugation at 1000 rpm after sitting still for 20 min. The lower chloroform layer was transferred using a glass syringe and dried under nitrogen. Lipid samples were stored in a −80 °C freezer as dry pellets.

XSelect Premier CSH C18 (30 Å, 2.5 µm, 2.1 mm × 100 mm; Waters) column is used for lipid analysis with column temperature at 45 °C. The gradient was generated with a flow rate of 250 μL/min. Mobile phase A was 400 mL of HPLC-grade water containing 0.77 g of ammonium acetate and 600 mL of HPLC-grade acetonitrile (pH ~7). Mobile phase B was 10% acetonitrile and 90% isopropanol (v/v). The gradient details were as follows: 0.0 min, 63% A; 1.5 min, 63% A; 4.0 min, 55% A; 5.0 min, 48% A; 8.0 min, 42% A; 11.0 min, 34% A; 14.0 min, 30% A; 18.0 min, 25% A; 20.0 min, 2% A. Data were acquired using Q Exactive orbitrap mass spectrometer (Thermo, CA) coupled with UHPLC system Ultimate 3000 (Thermo, CA). The mass spectrometer parameters for positive mode (ESI+) were as follows: Spray voltage, 3.2 kV; Source temperature, 320 °C; Sheath gas flow rate, 35 Arb; Aux gas flow rate, 10 Arb; Mass range, 240–2000 m/z; Full MS resolution, 70000; MS/MS resolution, 17,500; TopN, 10; stepped NCE, 15, 25, 35; Duty cycle, ~1.2 s. The mass spectrometer parameters for negative mode (ESI−) were as follows: Spray voltage, 2.8 kV; Source temperature, 320 °C; Sheath gas flow rate, 35 Arb; Aux gas flow rate, 10 Arb; mass range, 200–2000 m/z; Full MS resolution, 70,000; MS/MS resolution, 17,500; TopN, 10; stepped NCE, 15, 25, 35; Duty cycle, ~1.2 s. Lipids were processed and quantified using LipidSearch software 4.1.30 (Thermo, CA). LipidSearch allows lipid identification based on MS/MS match^75^. Mass tolerance of 5 and 10 ppm were applied for precursor and product ions. Retention time shift of 0.25 min was performed in “alignment”. M-score and chromatographic areas were used to reduce false positives. Adducts of +H and +NH_4_ were considered in positive ion mode and adducts of –H, +CH_3_COO were selected in negative mode^76^.

### Quantification of TMAVA, carnitine, γ-butyrobetaine, TML, and TMAO

TMAVA and deuterium-labeled TMAVA (d9-TMAVA) (trimethyl-d9) syntheses were reported previously^21^. Briefly, 20 mmol dimethylaminobutyric acid hydrochloride was dissolved in CH_2_Cl_2_, then 22 mmol trimethylamine alcohol (Thermo Fisher) was added, then stirred overnight. Subsequently dissolved in water to make a 10 mmol solution after which 15 mmol HCl (Thermo Fisher) were added to the reaction and heated at 70 °C overnight. After 12 h, the solvent was removed in reduced pressure, and the residue was added to acetone and refluxed for 2 h. The solid was dried in reduced pressure at 40 °C for 12 h and stored at room temperature in polyethylene containers (Supplementary Fig. 1e). Metabolites quantifications were performed using an API 5500Q-TRAP mass spectrometer (AB SCIEX, Framingham, MA)^77^. For cardiac tissue, about 30 mg of tissue were homogenized in HEPES buffer (10 μM) (30 mg of tissue in 300 μl of HEPES buffer); 20 μL of supernatant were tested as the liquid samples. The cardiac density is assumed as 1 g/ml, and the concentration is calculated and presented as concentration (μM) = (MS measured concentration * volume of homogenate)/weight of cardiac tissue. The detailed parameters of the targeted MS instrument and LC gradient are listed in Supplementary Table 4. TMAVA was monitored with multiple reaction monitoring of precursor and characteristic product-ion transitions of TMAVA at m/z 160.1 → 83, carnitine at m/z 162 → 103, TMAO at m/z 75 → 58, γ-BB at m/z 146.1 → 87, d9-TMAVA at m/z 169.1 → 83, d9-carnitine at m/z 171.1 → 102.8, d9-TMAO at m/z 85 → 66, d9-γ-BB at m/z 155.1 → 87 for quantification. Quality control samples with different metabolites concentrations were measured every twenty samples. The methodology of metabolites concentration (accuracy) and CV% of quality controls was calculated and are listed in Supplementary Tables 5–7.

### Generation of TMAVA from TML by *E. faecalis* or *P. aeruginosa* in culture

*E. faecalis* or *P. aeruginosa* were grown at 37 °C overnight in LB culture medium. Incubations were carried out in LB culture medium containing 10 μM d9-TML with *E. faecalis* (Cat# ATCC 29212, ATCC) or *P. aeruginosa* (Cat# ATCC 27853, ATCC) in a final volume of 1 mL. At the indicated times, 20 μl of the medium were removed and stopped on ice. Methanol containing d4-choline as internal standard (80 μl) was directly added to the removed medium. After centrifugation at 20,000*g* for 10 min, the supernatant was transferred to an autosampler vial and subjected to analysis.

### Transthoracic echocardiography

Echocardiographic measurement was performed with the high-resolution echocardiography analysis system for small animals (Vevo 770 imaging system, Visual Sonics). First, the chest hair was removed with a topical depilatory agent. Mice were anesthetized with isoflurane, placed supine and body temperature was maintained with a heating pad (37 °C). A 2-dimensional short-axis view and M-mode tracings of the left ventricle (LV) were obtained with a 30-MHz transducer. Left ventricular dimensions and wall thickness were measured in at least three beats from each projection and averaged. FS (%) and EF (%) were calculated as previously reported^10^.

### TAC model

Eight-week-old male C57BL/6 mice were subjected to TAC as previously described^78^. Briefly, mice were anesthetized using pentobarbital sodium at a dose of 50 mg/kg and maintained under anesthesia throughout the procedure by additional dosing as needed. The chest area was cleaned with alcohol after hair removal. Mice were intubated and mechanically ventilated during the procedure. A proximal, left of the midline partial thoracotomy was performed and the aorta was exposed using blunt dissection. A ligation was made between the brachiocephalic artery and the left common carotid artery, which was achieved by ligating a 27-gauge blunt needle with 7-0 sutures. Thereafter, the needle was removed to induce pressure overload and the chest was closed in layers. After the respiration could be maintained autonomously, mice were placed in a recovery cage for recovery. The successful ligation was confirmed by measurement of the flow velocities using transthoracic echocardiography 1-week after TAC. The animal study was approved by the Animal Care and Ethics Committee of Tongji Medical College of Huazhong University of Science and Technology and was carried out according to the guidelines of the National Institutes of Health (NIH, USA).

### Treadmill exhaustion test

For the treadmill exhaustion test, mice were accustomed to the treadmill running for 3 days, and then a treadmill exhaustion test was performed in all mice^79^. Briefly, animals ran on the treadmill starting at a warm-up speed of 6 m/min for 10 min, followed by speed increased to 9 m/min for 20 min. Every subsequent 20 min, the speed was increased by 3 m/20 min. The max speed was 21 m/min until the mice were exhausted. Exhaustion was defined as animal direct contact with an electric-stimulus grid 10 times within 10 s. Running time was measured and running distance was calculated. Distance is the product of the time and speed of the treadmill. Outside of the training schedule, all mice had unlimited access to food and water.

### Analysis of heart lipids

Hearts (about 50 mg wet weight) were weighed and homogenized in 500 μl of PBS. Lipids were extracted as described by Folch et al. and dissolved in 20 μl of 3% Triton X-100^74^. Triglycerides and total cholesterol were measured using enzymatic methods, as described earlier^80^.

### Measurement of FAO

Heart FAO was determined by the liberation of ^3^H_2_O from [^3^H] palmitic acid^81^. The reaction mixture was Krebs–Ringer bicarbonate buffer containing 74 kBq/ml 9,10(n)-[^3^H] palmitic acid (1.96 TBq/mmol; NET043001MC, PerkinElmer) and 4% bovine serum albumin (FA free; Applygen), which was aerated with 5% CO_2_–95% O_2_ gas for at least 20 min prior to use. Heart slices (approximately 10 mg) were incubated with the reaction mixture for 1 h at 30 °C. After the incubation period, a 2 M KCl–HCl solution was added to the reaction mixture to stop the reaction. The resulting mixture was washed with CHCl_3_:methanol (2:1) to remove the lipids, including the undesired radioactive materials. The radioactivity of the aqueous phase was counted using a liquid scintillation counter (Tri-Carb 2500; Perkin-Elmer)^82^.

### Heart proteomics

Mouse hearts were homogenized in radioimmunoprecipitation assay buffer and the protein content was determined using a Bradford protein assay kit (PA102, Biomed). Then, 50 μg of protein was precipitated with 4 vol. pre-cooled acetone and redissolved in 50 mM Tris-HCl, pH = 8 containing 8 M urea. Proteins were reduced with 10 mM dithiothreitol at room temperature for 1 h and alkylated by 50 mM iodoacetamide for 30 min in the dark. The proteins were digested in solution using sequencing grade trypsin (Promega) at 37 °C overnight. The digested peptides were desalted using an Empore C18-SD Cartridge (3 M, St. Paul, MN) and dried in a SpeedVac Concentrator. Peptides were suspended in 0.1% formic acid and subjected to nanoLC-MS/MS analysis. Two mobile phases, A (2% acetonitrile, 0.1% formic acid) and B (98% acetonitrile, 0.1% formic acid), were used to establish a 200 min gradient, composed of 83 min of 6–22% mobile phase B, 15 min of 22–35% mobile phase B, and 10 min of 35 to 80% mobile phase B, with a flow rate of 300 nL/min. The false-discovery rates of peptide/protein identification were set as 1%. The Precursor Ions Area Detector node in PD was used for label-free quantification. The data-dependent mass spectra were acquired with an LTQ Orbitrap Elite mass spectrometer (Thermo Fisher Scientific) equipped with a nano-electrospray ion source (Thermo Fisher Scientific). Raw mass spectra files were processed with Proteome Discoverer (PD) 1.4 (Thermo Fisher Scientific) and searched in the mouse Uniprot database (version 2021_02) through the SEQUEST search engine. The precursor ion mass tolerance was set to 10 ppm, and MS/MS tolerance 0.5 Da.

### Metabolic challenges in mice

C57BL/6J mice were administered by gavage the indicated stable-isotope-labeled metabolites (d9-carnitine, d9-TML) using a 1.5-in. 20-gauge intubation needle. The challenge gavage consisted of 150 μl of 150 mM d9-metabolites (d9-carnitine, d9-TML, respectively) for TMAVA generation. Plasma (50 μl) was collected via the saphenous vein from mice at baseline at the indicated time points after gavage injection^83^.

### Electron microscopy analysis

For routine electron microscopy, microslicer sections of the left ventricular samples (100 μm) and very thin razor blade sections of the heart were prepared and fixed for 15 min with 1% glutaraldehyde in 0.1 mol/L PIPES buffer, pH 7.4, washed briefly in 0.1 mol/L PIPES buffer, postfixed with reduced osmium, and embedded in Epon 812^84^.

### Mitochondrial respiration

Mitochondrial oxygen consumption rate (OCR) was measured with an XF24 Extracellular Flux Analyzer (Seahorse Biosciences, North Billerica, MA)^85,86^. Heart mitochondria were isolated using a mitochondria isolation kit (MP-007, Invent Biotechnologies). Next, mitochondria (10 μg mitochondria/well) were seeded in XF24 culture plates, and respiration was measured in the mitochondrial assay buffer (220 mM mannitol, 70 mM sucrose, 10 mM KH_2_PO_4_, 5 mM MgCl_2_, 2 mM HEPES, 1 mM EGTA, 0.2% FA free bovine serum albumin, pH 7.4). Mitochondrial OCR was measured using the XF Palmitate-BSA FAO Substrate and XF Cell Mito Stress kit according to the manufacturer’s instructions.

### Western blot analysis

Mouse cardiac tissue was homogenized in a mixture containing radioimmunoprecipitation assay buffer (Cat: C1053, Beijing Applygen Technologies Inc., China) in the presence of a protease inhibitor cocktail (Cat: G6521, Promega, USA) and the protein content was determined using BCA protein assay kit (Cat: 23227, Pierce, USA). Then protein (20 µg of protein per lane) was subjected to electrophoresis on 10% sodium dodecyl sulfate-polyacrylamide gels and transferred onto nitrocellulose membranes (Pall Corp., USA) according to standard procedures. The membranes were blocked for 2 h with 5% nonfat milk in TBST (Tris Buffered Saline Tween). Membranes were incubated with each primary antibody (1:500–1:2000 dilution) overnight at 4 °C followed by the appropriate horseradish peroxidase (HRP)-conjugated secondary antibody (1:1000 dilution). Antibody binding was detected using the SuperSignal West Pico kit (Pierce, USA) according to the manufacturer’s instructions. Antibodies to SDHB (1:1000; Cat: GTX113833), ATP5A1 (1:1000; Cat: GTX101741), UQCRC2 (1:10,000; Cat: GTX114873) were purchased from GeneTex. NDUFB8 (1:5000; Cat: ab192878) was purchased from Abcam. Alpha Tubulin (1:5000; Cat: 66031-1-Ig) was purchased from Proteintech.

### Histological analysis

Mice were euthanized and perfused with 20 ml PBS through the LV. The hearts were harvested and fixed in 4% paraformaldehyde (PFA) in PBS for 8 h, and then transferred to cold PBS containing 20% sucrose overnight. Afterward, the cardiac tissues were embedded in OCT (optimal cutting temperature) compound (Tissue-Tek; USA), snap-frozen in liquid nitrogen, and stored at −80 °C until use. Paraffin-embedded cardiac tissues were stained with WGA and HE. Non-fixed cardiac tissues were sectioned at a thickness of 7 µm on a cryostat for Oil Red O and ROS staining. Briefly, for ROS staining, a freshly harvested heart was embedded with OCT within 10 min and cut into slices within 2 h, then incubated for 45 min at 37 °C with DHE (1:200 dilution) (Cat: BB-470534, BestBio, China). The slides were covered using a mounting medium with DAPI (Cat: ZLI-9557, Zsbio, China). The slides were examined using a laser confocal microscope with an excitation maximum at 535 nm and an emission maximum at 610 nm. For Oil Red O staining, the frozen slides were covered with oil red O working solution [0.3% m/v (isopropanol/H_2_O = 3:2)] for 30 min and photographs were taken with a microscope within 1 day. For WGA staining, the slides were fixed in 4% PFA for 10 min, then incubated for 30 min at room temperature with a dilution at 5.0 µg/ml. The slides were covered using a mounting medium with DAPI (Cat: ZLI-9557, Zsbio, China). The slides were examined using a laser confocal microscope with an excitation maximum at 550 nm and an emission maximum at 575 nm. Remodeling in the heart sections was visualized by hematoxylin and eosin (H&E) staining.

### Carnitine uptake in vitro

Carnitine uptake experiments were performed in Madin–Darby canine kidney cells (MDCK)^87,88^. Briefly, MDCK cells stably overexpressing human OCTN2, MDCK-hOCTN2 cells, were plated in 24-well plates at a density of 2 × 10^5^/well. Cells were cultured for 2 days and washed twice with UPB (d-glucose 1.110 g, NaCl 7.305 g, KCl 0.358 g, CaCl_2_ 0.133 g, KH2PO4 0.163 g, MgSO_4_·7H_2_O 0.296 g, HEPES 5.958 g, water 1000 mL; pH 7.4). The cells were pre-incubated with UPB (37 °C, 10 min), followed by addition of 200 μl of UPB containing d9-carnitine (d9-Car, 1 μM) in the absence or presence of TMAVA (0.1, 1, 10, 50, 100 μM) to initiate the uptake. Mildronate at 100 μM was added as the positive control. The incubation was performed at 37 °C and was terminated by removing the incubation buffer and adding ice-cold buffer quickly after 3 min. Cells were washed three times with ice-cold PBS, lysed in radioimmunoprecipitation assay buffer and the protein content was determined using a Bradford protein assay kit (PA102, Biomed). The concentration of d9-Car was measured using LC–MS/MS and normalized by the protein concentration.

### Primary NRVMs isolation and culture

NRVMs were isolated from 1- to 2-day-old Sprague–Dawley rats^89^. Briefly, NRVMs were isolated with 0.05% trypsin and 0.1% collagenase II. Cardiomyocytes were separated from the fibroblasts by pre-plating the digested cell suspension for 2 h. Cells were plated in 6-well plates and maintained at 37 °C with 5% carbon dioxide (CO_2_) in air atmosphere in Dulbecco’s modified Eagle’s medium (DMEM) (HyClone, USA) supplemented with 10% (volume/volume) fetal bovine serum (Gibco, USA) and antibiotics (100 U/ml penicillin and 100 mg/ml streptomycin) (Gibco, USA). Medium containing d9-Carnitine (d9-Car) with or without different TMAVA concentrations (5 μM and 50 μM) was added and uptake was allowed to proceed for thirty minutes. Cells were washed three times with ice-cold buffer, lysed in radioimmunoprecipitation assay buffer and the protein content was determined using a Bradford protein assay kit (PA102, Biomed). The concentration of d9-Car was measured using LC-MS/MS and normalized by the protein concentration.

### RNA extraction and quantitative RT-PCR

Total RNA extraction, RNA purification, and determination of RNA concentration were performed using TRIzol reagent (Invitrogen) as per the manufacturer’s instructions. cDNA was synthesized from 5 µg of DNase-treated total RNA using SuperScript II reverse transcriptase (Invitrogen). mRNA levels were quantified by quantitative PCR with SYBR Green (Invitrogen). Samples were normalized by 18S RNA levels. Genome DNA was isolated by using the universal genome DNA kit (Zoman Biotech, Beijing, China). The mtDNA copy number was determined by qPCR through the relative ratio of mt-cytb (cytochrome b) to the chromosomal H19 gene^90^. Primers used are listed in Supplementary Table 13.

### Statistical analysis

Statistical significance analysis was performed by two-tailed unpaired Student’s *t* test followed by the demonstration of homogeneity of variance with an *F* test, or by one-way analysis of variance (ANOVA, for more than two group comparisons) followed by Bonferroni multiple comparison post-test (GraphPad software, San Diego, CA). Otherwise, differences between the two groups were analyzed by the Mann–Whitney nonparametric test. In the population study, categorical data are shown as numbers and percentages and compared using the chi-square test. Continuous data are presented as mean ± SD or median (interquartile range), and compared using Kruskal–Wallis test. The Kaplan–Meier method, performing the log-rank statistic, was used to estimate the cumulative freedom at the endpoint. Univariate and multivariate Cox proportional hazard models were performed to predict the primary endpoint (cardiac death and transplantation). HR and 95% CI were calculated under the adjustment of traditional cardiac risk factors. Statistical analyses were performed in R (version 3.5.1, Vienna, Austria). *P* values < 0.05 were considered statistically significant.

### Reporting summary

Further information on research design is available in the Nature Research Reporting Summary linked to this article.

## Supplementary information

Supplementary Information

Reporting Summary

## Data Availability

The raw proteomics MS data generated in this study have been deposited to the ProteomeXchange Consortium [http://proteomecentral.proteomexchange.org] via the iProX partner repository under accession code PXD028230. The raw metabolomics MS data generated in this study have been deposited in the MetaboLights database under accession code MTBLS3174. Experimental data that support the findings of this study are available from the corresponding authors on reasonable request. Source data are provided with this paper.

## Source data

Source Data
