# Supplementary material for: Gut microbiota production of trimethyl-5-aminovaleric acid reduces fatty acid oxidation and accelerates cardiac hypertrophy
Source: Nat Commun. 2022 Apr 1;13:1757. doi: 10.1038/s41467-022-29060-7 (PMC8976029; doi:10.1038/s41467-022-29060-7)
Supplement: Supplementary file 1 — Supplementary Information [file 41467_2022_29060_MOESM1_ESM.pdf]

Supplementary Information

Gut microbiota production of trimethyl-5-aminovaleric acid reduces fatty acid oxidation and accelerates cardiac hypertrophy

Mingming Zhao,[1,2#] Haoran Wei,[3#] Chenze Li, [4] Rui Zhan,[2] Changjie Liu,[2] Jianing Gao,[2] Yaodong Yi,[5] Xiao Cui,[6] Wenxin Shan,[2] Liang Ji,[2] Bing Pan,[2] Si Cheng,[7] Moshi Song,[8] Haipeng Sun,[9] Huidi Jiang,[5] Jun Cai,[10] Minerva T. Garcia-Barrio,[11] Y. Eugene Chen,[11] Xiangbao Meng,[12] Erdan Dong, [1,2] Dao Wen Wang,[3*] Lemin Zheng[2, 7*]

[#] Mingming Zhao and Haoran Wei contributed equally to this work.

[1] Department of Cardiology and Institute of Vascular Medicine, Peking University Third Hospital, Beijing, China.

[2] The Institute of Cardiovascular Sciences and Institute of Systems Biomedicine, School of Basic Medical Sciences, Key Laboratory of Molecular Cardiovascular Science of Ministry of Education, NHC Key Laboratory of Cardiovascular Molecular Biology and Regulatory Peptides, Beijing Key Laboratory of Cardiovascular Receptors Research, Health Science Center, Peking University, Beijing 100191, China.

[3] Division of Cardiology, Department of Internal Medicine and Hubei Key Laboratory of Genetics and Molecular Mechanisms of Cardiological Disorders, Tongji Hospital, Tongji Medical College, Huazhong University of Science and Technology, Wuhan 430030, China.

[4] Department of Cardiology, Zhongnan Hospital of Wuhan University, Wuhan, 430071, China;

[5] Laboratory of Pharmaceutical Analysis and Drug Metabolism, College of Pharmaceutical Sciences, Zhejiang University, Hangzhou 310058, China

[6] Department of Cardiology, The First Affiliated Hospital, College of Medicine, Zhejiang University, Hangzhou, China

[7] Beijing Tiantan Hospital, China National Clinical Research Center for Neurological Diseases, Advanced Innovation Center for Human Brain Protection, The Capital Medical University, Beijing 100050, China.

[8] State Key Laboratory of Membrane Biology, Institute of Zoology, Chinese Academy of Sciences, Beijing, 100101, China.

[9] Department of Pathophysiology, Key Laboratory of Cell Differentiation and Apoptosis of Chinese Ministry of Education, Shanghai Jiao Tong University School of Medicine, Shanghai, 200025, China.

[10] Fuwai Hospital, State Key Laboratory of Cardiovascular Diseases, National Center for Cardiovascular Diseases, Chinese Academy of Medical Sciences and Peking Union Medical College, Beijing, China.

[11] Cardiovascular Center, Department of Internal Medicine, University of Michigan Medical Center, Ann Arbor, MI 48109, USA.

[12] State Key Laboratory of Natural and Biomimetic Drugs, School of Pharmaceutical Sciences, Peking University, Beijing 100191, China.

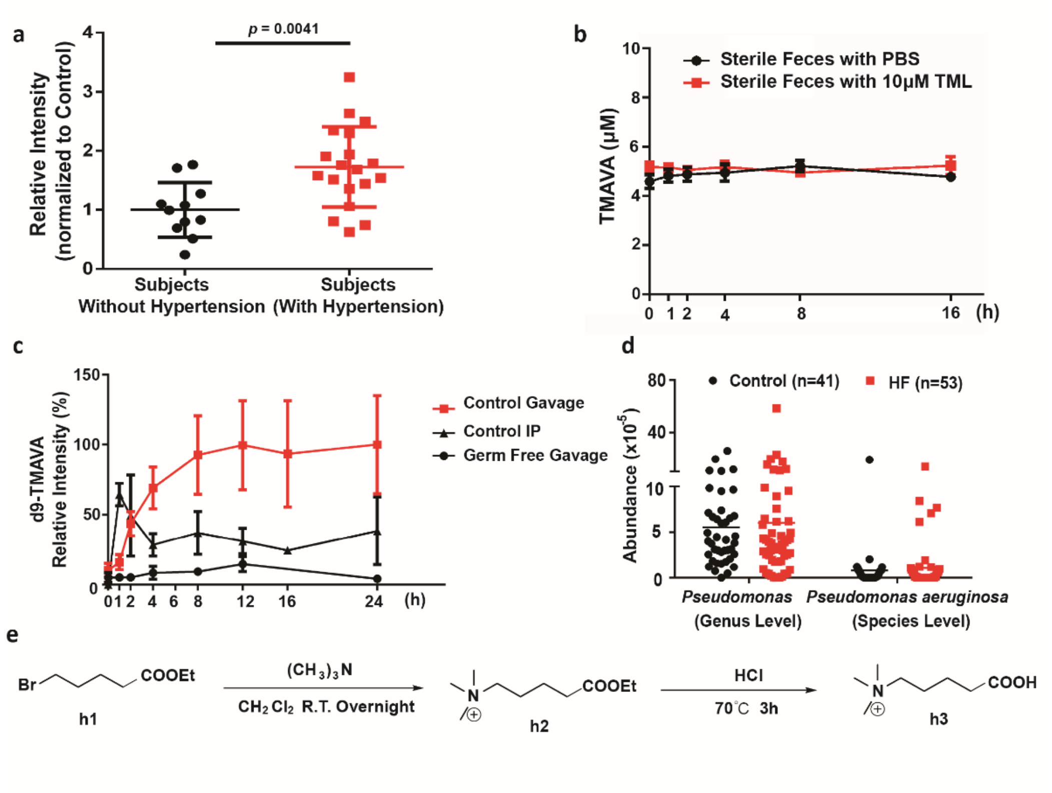

Supplemental Figure 1. TMAVA is generated by gut microbiota. a, Relative abundance of TMAVA in plasma from patients with hypertension compared with controls based on untargeted global metabolomics (n=11 and 19). **b,** Feces from conventionally raised mice were sterilized, co-incubated with 10μM TML and TMAVA levels were measured. **c,** Conventional mice (control) and germ free mice were gavaged with d9-TML. Conventional mice were intraperitoneal injected with d9-TML (control IP). Post-challenge measurements of d9-TMAVA were performed in serial venous blood draws at the indicated times by stable isotope dilution LC-MS. **d,** *Pseudomonas* at the genus level and *P. aeruginosa* at the species level were analyzed in patients using metagenomic sequencing. **e,** Flow chart for the synthesis of TMAVA. Statistical significance was evaluated by two-tailed unpaired Student's t test [(a)] or two-tailed nonparametric Mann-Whitney test [(d)] [*P < 0.05, **P < 0.01,

***P < 0.001]. Data are expressed as mean ± SEM. Source data are provided as a Source Data file.

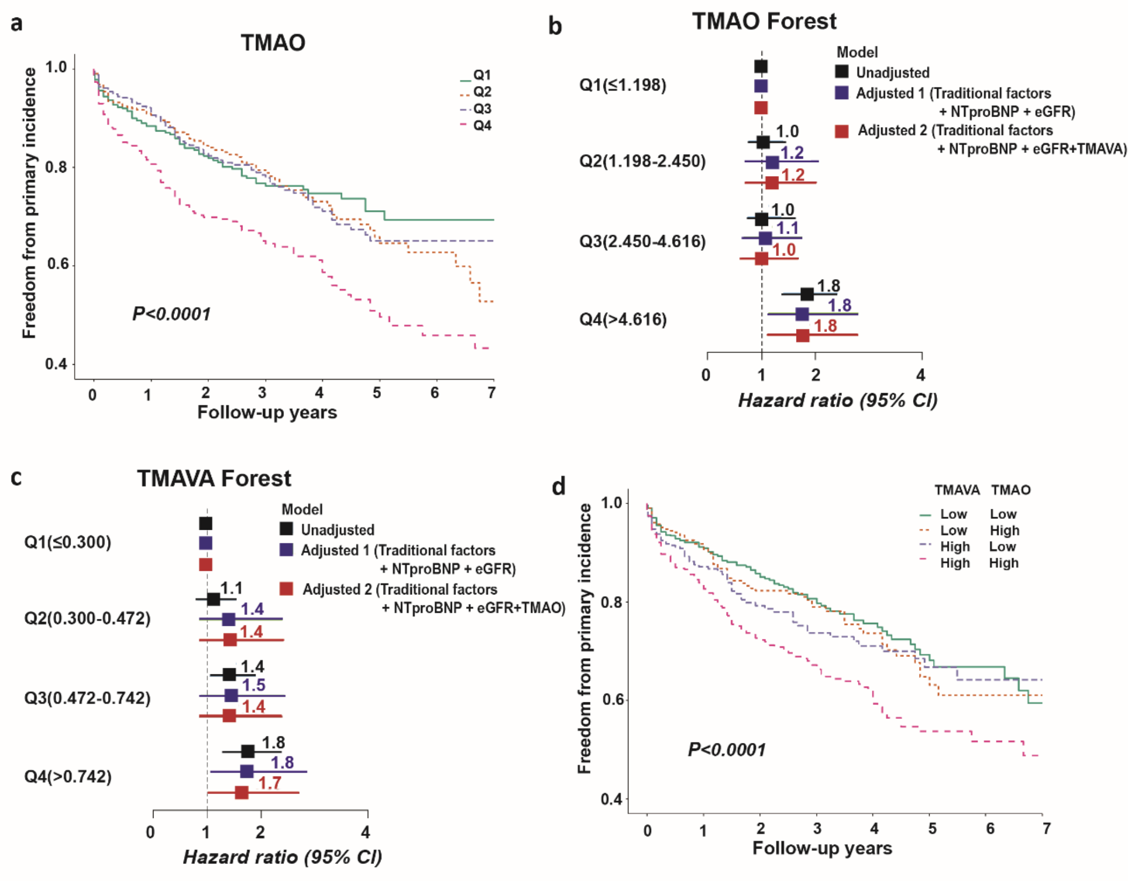

Supplemental Figure 2. Elevated plasma TMAO level is associated with incident cardiac death and transplantation risk, independent of traditional risk factors.

a, Kaplan-Meier estimate of 7-year risk for cardiac death and transplantation ranked by TMAO quartiles in the cohort (N=1647). **b,** Forest plots indicate the HR (95% CI) for incident (7-year) risk for cardiac death and transplantation according to TMAO quartiles. HR (unadjusted, black squares) and multivariate Cox model 1 adjusted (blue squares; adjusted for age, sex, smoking, SBP, diabetes, high-density lipoprotein [HDL], low-density lipoprotein [LDL], eGFR and NTproBNP), or model 2 adjusted (red squares, adjusted for model 1 and TMAVA). The 95% confidence interval is indicated by the line length. **c,** Forest plots indicate the HR (95% CI) for incident (7-year) risk for cardiac death and transplantation according to TMAVA quartiles. HR

(unadjusted, black squares) and multivariate Cox model 1 adjusted (blue squares; adjusted for age, sex, smoking, SBP, diabetes, high-density lipoprotein [HDL], low-density lipoprotein [LDL], eGFR and NTproBNP), or model 2 adjusted (red squares, adjusted for model 1 and TMAO). The 95% confidence interval is indicated by the line length. **d,** Kaplan–Meier plot illustrating the relationship between plasma TMAO and incident 7-year risk for mortality according to TMAVA and TMAO levels, where each marker is categorized above vs. below the median level in the cohort. Median plasma concentration of TMAVA and TMAO within the cohort were used to stratify patients as ‘high’ (⩾median) or ‘low’ (<median) values; also shown is hazard ratio (95% confidence interval) for the indicated TMAVA and TMAO group. Source data are provided as a Source Data file.

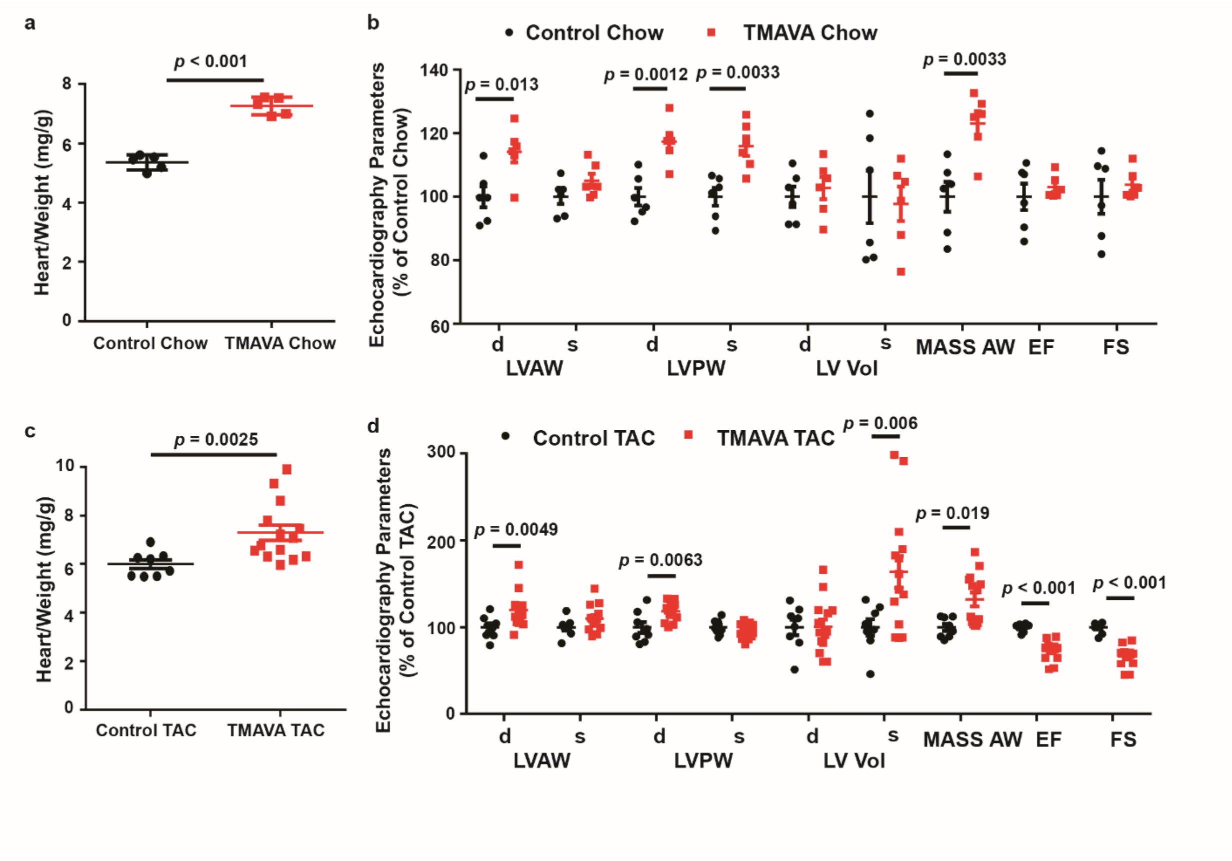

Supplemental Figure 3. TMAVA induced cardiac hypertrophy in mice on a chow diet (CD) or transverse aortic constriction (TAC)-induced LV pressure overload. Mice were fed a CD for 12 weeks, with the TMAVA group treated with 0.325 % TMAVA (m/v %) in the drinking water in [(a)-(b)], n=5-6/group. **a,** The ratio of heart weight to body weight was significantly increased in the TMAVA-treated mice (n=5/group). **b,** Echocardiographic assessment showed that left ventricular anterior wall (LVAW), posterior wall (LVPW) thickness and LV mass were increased in the TMAVA-treated mice on CD (n=6/group). Mice at 6 weeks of age, fed a CD, were pre-treated with 0.325 % TMAVA (m/v %) in the drinking water for 2 weeks in the TMAVA group. Then mice were subjected to transverse aortic constriction (TAC) procedures in [(c)- (d)], n=8 and 14. **c,** The ratio of heart weight to body weight were

significantly increased in the TMAVA-treated mice with TAC procedure. **d,** Echocardiographic assessment showed that left ventricular anterior wall (LVAW), posterior wall (LVPW) thickness and LV mass were increased in the TMAVA-treated mice subjected to the TAC procedure. EF and FS were decreased. Abbreviations: d, diastolic; s, systolic; LVAW, left ventricular anterior wall; LVPW, left ventricular posterior wall; AW, anterior wall; EF, ejection fraction; FS, shortening fraction. Statistical significance was evaluated by two-tailed unpaired Student's t test [(a) - (c)] or two-tailed nonparametric Mann-Whitney test [(d)] [*P < 0.05, **P < 0.01, ***P < 0.001]. Data are expressed as mean ± SEM. Source data are provided as a Source Data file.

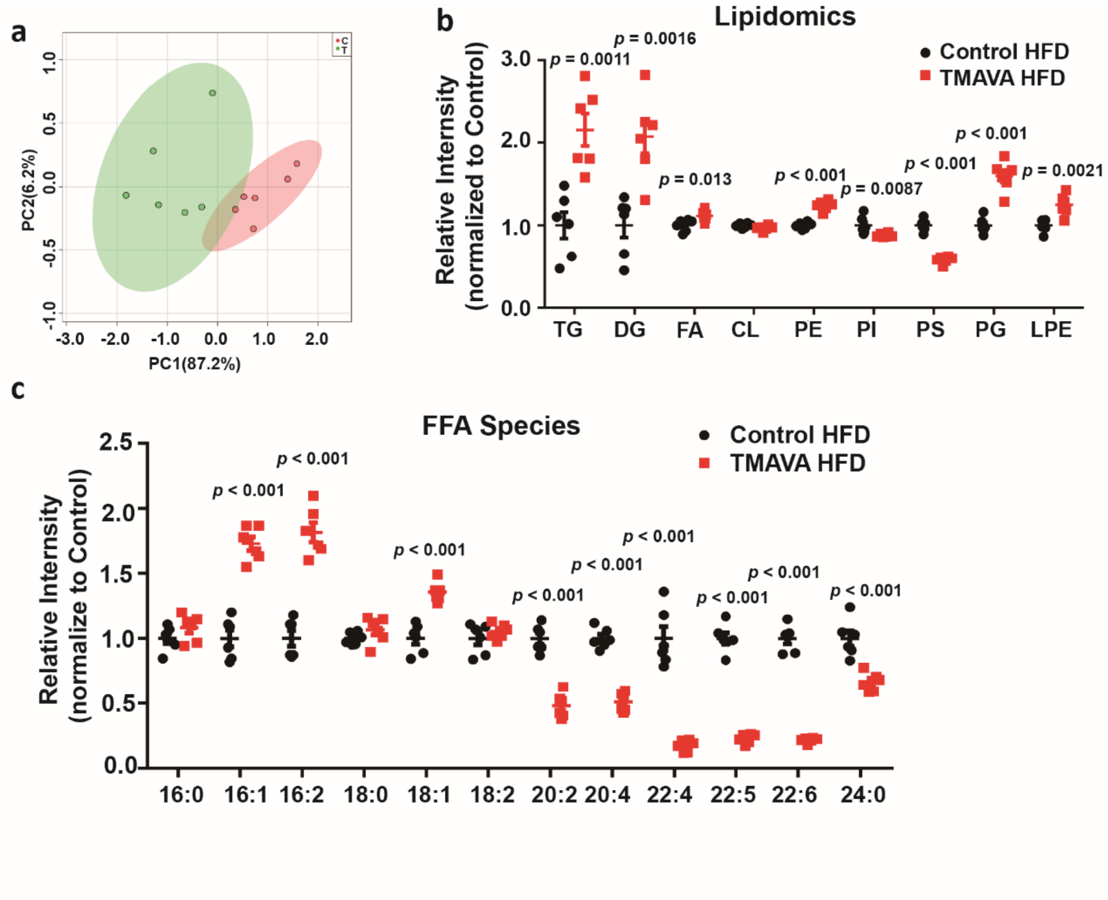

Supplemental Figure 4. TMAVA treatment leads to cardiac lipid accumulation and lipid metabolism dysfunction. Mice (n=6/group) were fed a HFD for 12 weeks, with the TMAVA group treated with 0.325 % TMAVA (m/v %) in the drinking water in [(a)-(c)]. **a,** Comparative myocardial lipidomics of the two groups as described in the methods. Principal component analysis (PCA) score plot of cardiac lipid profiling in 6 controls and 6 TMAVA-treated mice on HFD (n=6/group). **b,** Different cardiac lipid species were analyzed in control and TMAVA-treated mice on HFD (n=6/group). TG, triacylglycerol; DG, diglyceride; FA, fatty acid; PC, phosphatidylcholine; PI, phosphatidylinositol; LPC, lysophosphatidylcholine; LPE, lysophosphatidylethanolamine; LPI, lysophosphatidylinositol, and PG, phosphatidylglycerol. **c,** Free fatty acid (FFA) species were measured in control and

TMAVA-treated mice on HFD (n=6/group). Statistical significance was evaluated by two-tailed unpaired Student's t test [(c)] or two-tailed nonparametric Mann-Whitney test [(b)] (*P < 0.05, **P < 0.01, ***P < 0.001). Data are expressed as mean ± SEM. Source data are provided as a Source Data file.

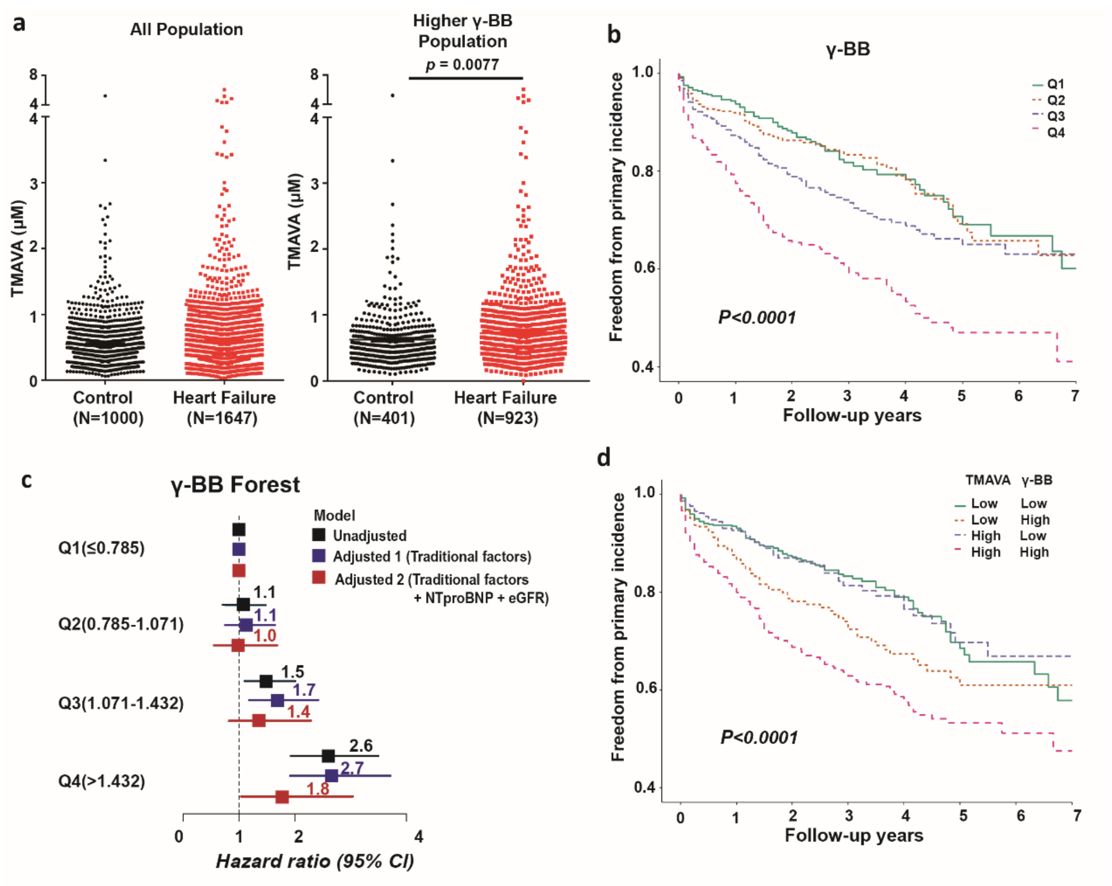

Supplemental Figure 5. Elevated plasma γ-butyrobetaine (γ-BB) level is associated with incident cardiac death and transplantation risk, independent of traditional risk factors. a, TMAVA in plasma from patients with heart failure compared with controls in the whole population (left) and in the higher γ-BB sub-population (right) based on targeted metabolomics. **b,** Kaplan-Meier estimate of 7-year risk for cardiac death and transplantation ranked by γ-BB quartiles in the cohort (N=1647). **c,** Forest plots indicate the HR (95% CI) for incident (7-year) risk for cardiac death and transplantation according to γ-BB quartiles. HR (unadjusted, black squares) and multivariate Cox model 1 adjusted (blue squares; adjusted for age, sex, smoking, SBP, diabetes, high-density lipoprotein [HDL], low-density lipoprotein [LDL]), or model 2 adjusted (red squares, adjusted for model 1 plus eGFR and

NTproBNP). The 95% confidence interval is indicated by the line length. **d,** Kaplan–Meier plot illustrating the relationship between plasma γ-BB and incident 7-year risk for mortality according to γ-BB and TMAVA levels, when each marker was categorized above vs. below the median level in the cohort. Median plasma concentrations of γ-BB or TMAVA within the cohort was used to stratify patients as 'high' (⩾median) or 'low' (<median) values, respectively. Statistical significance was evaluated by two-tailed nonparametric Mann-Whitney test [(a)] [**P < 0.01]. Data are expressed as mean ± SEM. Source data are provided as a Source Data file.

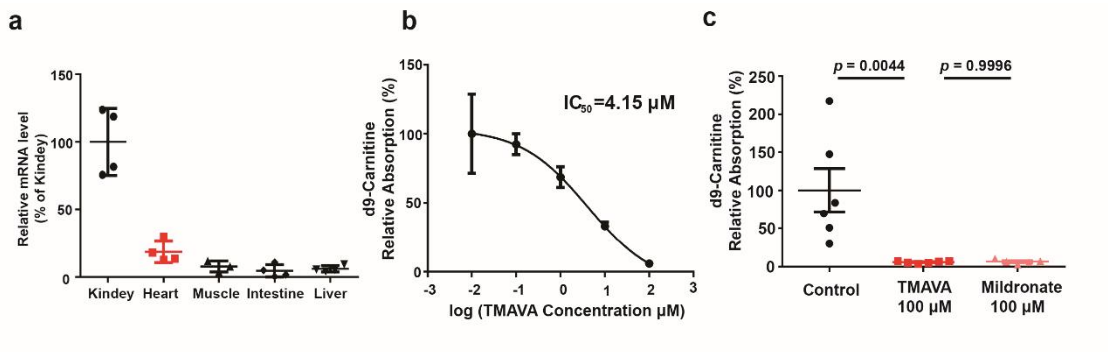

Supplemental Figure 6. TMAVA inhibited carnitine metabolism through BBOX and OCTN2 function. a, Relative basal OCTN2 mRNA levels in various tissues from mice in chow diet (n = 4/group). Expression level was determined by RT-qPCR and normalized to 18S expression. **b,** MDCK cells stably overexpressing hOCTN2 cloned in the pcDNA3.1 plasmid, were established by selection with G418. d9-carnitine uptake in MDCK-hOCTN2 cells was measured in the presence of various concentrations of TMAVA. TMAVA inhibited the uptake of d9-carnitine in the MDCK-hOCTN2 cells (n=6/concentration). **c,** d9-carnitine uptake in MDCK-hOCTN2 cells was measured in the presence of 100μM TMAVA or 100μM Mildronate (a positive control to inhibit carnitine transport) (n=6/group). Statistical significance was evaluated by one-way ANOVA [(c)] (*P < 0.05, **P < 0.01, ***P < 0.001). Data are expressed as mean ± SEM. Source data are provided as a Source Data file.

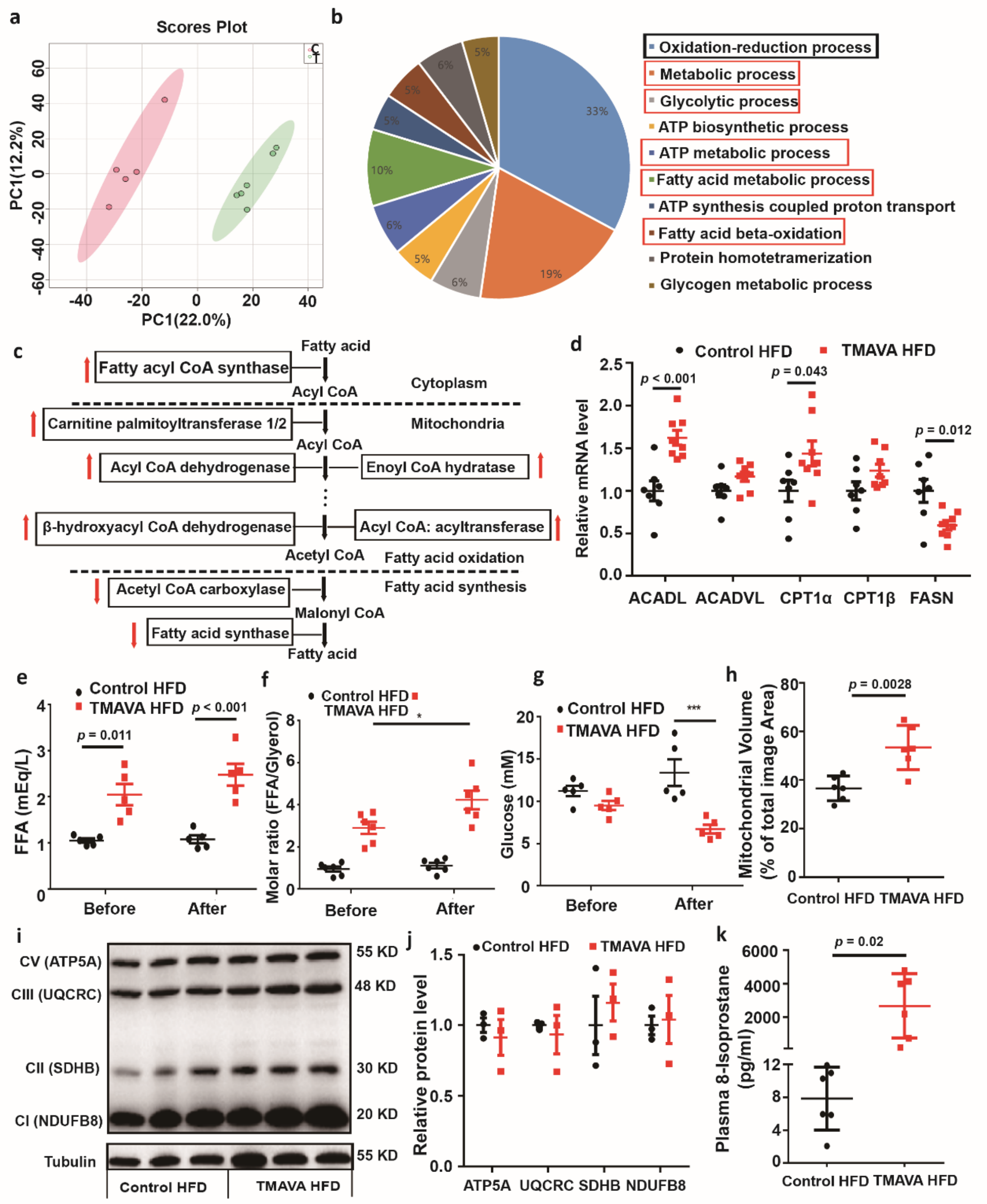

Supplemental Figure 7: TMAVA treatment results in perturbation of cardiac energy metabolism. Mice were fed a HFD for 12 weeks, with the TMAVA group treated with 0.325 % TMAVA (m/v %) in the drinking water in [(a)-(b) and (d)-(j)]. **a,** Comparative myocardial proteomics of the two groups as described in the methods.

Principal component analysis (PCA) score plot of myocardial protein profiling in 5 controls and 6 treated mice. **b,** Distribution among cellular biological processes of the proteins identified in the proteomic analysis. **c,** The pathway of FFA metabolism. **d,** RT-qPCR analysis of myocardial mRNA expression of lipid oxidation and lipogenic genes after TMAVA treatment (n=7 and 8). **e-g,** Control and TMAVA-treated mice fed a HFD for 12 weeks (n=5/group) were subjected to treadmill exhaustion test and their circulating FFA (**e**), FFA/glycerol molar ratio (**f**) and glucose levels (**g**) were measured. **h,** Mitochondria volume (% of total image area) was calculated with ImagJ. **i,** Western blot of mitochondrial complexes (CI subunit NDUFB8, CII subunit SDH, CIII subunit UQCRC and CV ATP5A) (n=3/group). **j,** Quantification results of the western blot (n=3/group). **k,** The plasma oxidative stress marker 8-isoprostane was measured after 12-weeks' treatment with HFD (n=6). Statistical significance was evaluated by two-tailed unpaired Student's t test [(d), (h) and (k)], one-way ANOVA [(e-g)] or two-tailed nonparametric Mann-Whitney test [(j)] [*P < 0.05, **P < 0.01, ***P < 0.001]. Data are expressed as mean ± SEM. Source data are provided as a Source Data file.

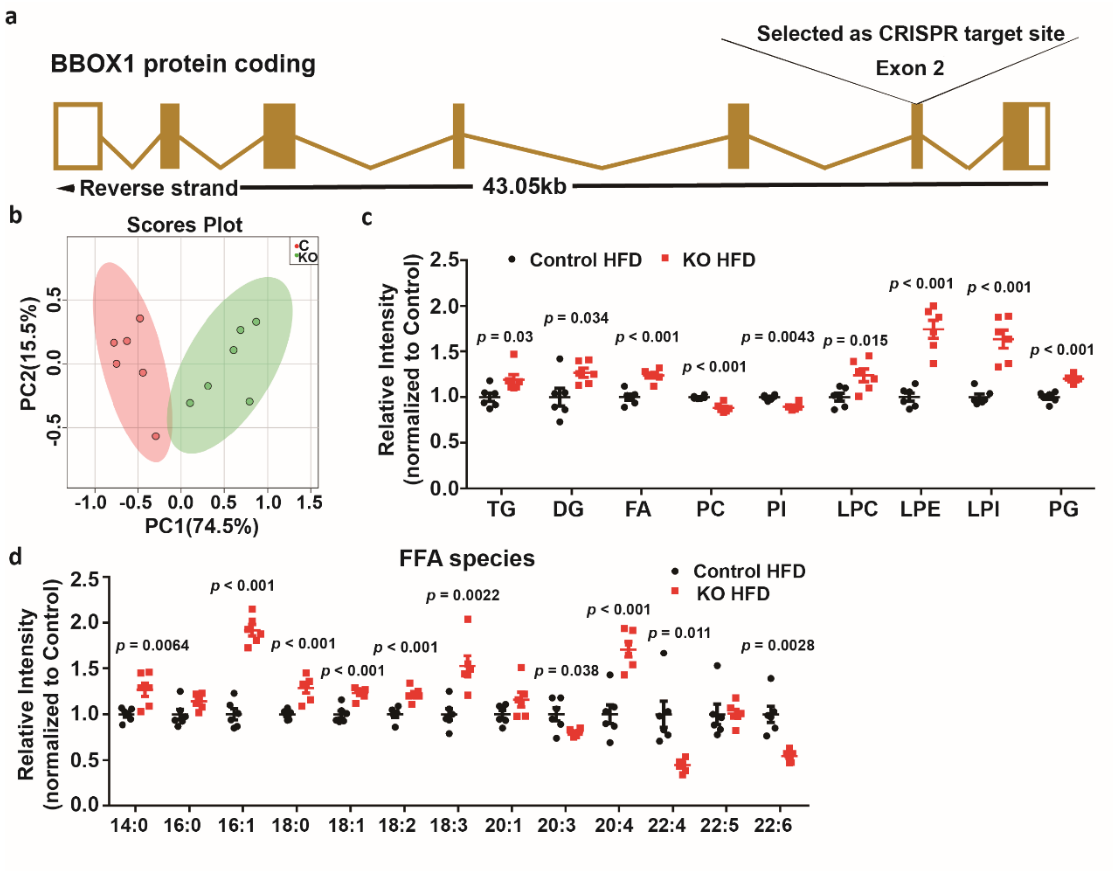

Supplemental Figure 8: BBOX deficiency leads to cardiac lipid accumulation. a, The BBOX knockout mice ($BBOX^{-/-}$) mouse line was created using CRISPR- Cas9 to introduce a 5bp deletion in the exon 2 of the BBOX gene. WT (control) and BBOX $^{-/-}$ mice were fed a HFD for 12 weeks in [(b)-(d)]. **b,** Comparative myocardial lipidomics of the two groups as described in the methods. Principal component analysis (PCA) score plot of cardiac lipid profiling in 6 controls and 6 $BBOX^{-/-}$ mice. **c,** Different lipid species were analyzed using lipidomics for the hearts from control and $BBOX^{-/-}$ mice described above (n=6/group). TG indicates triacylglycerol; DG, diglyceride; FA, fatty acid; PC, phosphatidylcholine; PI, phosphatidylinositol; LPC, lysophosphatidylcholine; LPE, lysophosphatidylethanolamine; LPI, lysophosphatidylinositol, and PG, phosphatidylglycerol. **d,** FFA species in control and

BBOX$^{-/-}$ mice (n=6/group). Statistical significance was evaluated by two-tailed nonparametric Mann-Whitney test [(c-d)] (*P < 0.05, **P < 0.01, ***P < 0.001). Data are expressed as the mean ± SEM. Source data are provided as a Source Data file.

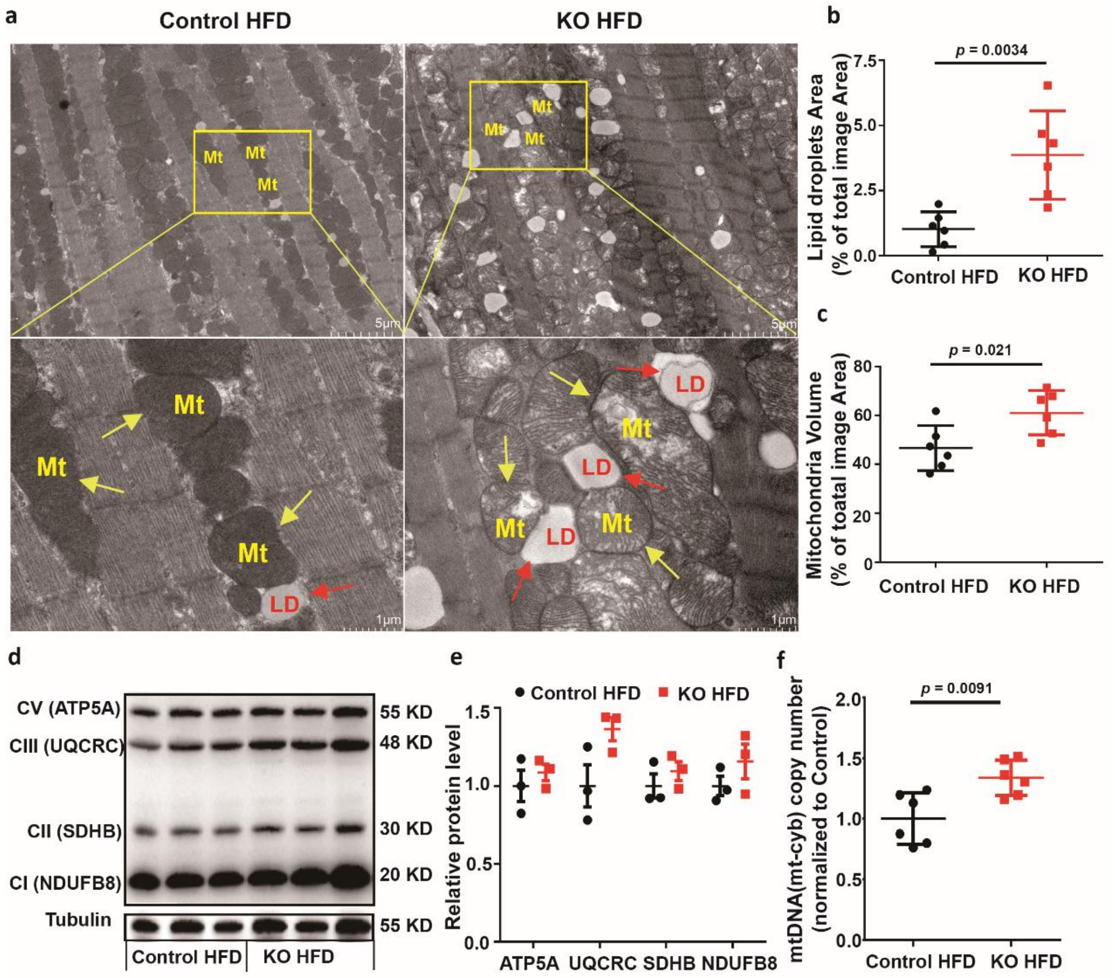

Supplemental Figure 9: BBOX knock out results in accumulation of dysfunctional mitochondria. WT (control) and BBOX knockout mice (BBOX$^{-/-}$) were fed a HFD for 12 weeks in [(a)-(f)] **a,** Transmission electron microscopy (TEM) of hearts from control and BBOX$^{-/-}$ mice fed a HFD for 12 weeks. Representative images of left ventricular TEM assessment. Mt, mitochondria (original magnification 10K ×). **b,** Lipid droplet percentage was calculated using ImagJ relative to total image area (n=6/group). **c,** Quantification results of mitochondrial volume (% of the total image area) of heart sections from TEM images (n=6/group). **d,** Western blot of mitochondrial complex CI subunit NDUFB8, CII subunit SDH, CIII subunit UQCRC

and CV subunit ATP5A (n=3/group). **e,** Quantification results of the western blot (n=3/group). **f**, RT-qPCR of mtDNA-encoded gene (mt-cytb) relative to nuclear-encoded H19 (n=6). Statistical significance was evaluated by two-tailed unpaired Student's t test [(b-c) and (f)] or two-tailed nonparametric Mann-Whitney test [(e)] (*P < 0.05, **P < 0.01, ***P < 0.001). Data are expressed as mean ± SEM. Source data are provided as a Source Data file.

Supplementary Table 1.

Baseline characteristics of subjects in the learning cohort.

	Subjects without Hypertension (n=11)	Subjects with Hypertension (n=19)
Male,%	54.5	31.6
Diabetes, %	45.5	57.9
Dyslipidemia, %	63.6	73.7
Liver steatosis, %	27.3	57.9
Total cholesterol, mmol/L	4.35 ± 1.11	4.17 ± 1.04
Triglycerides, mmol/L	1.82 ± 0.96	2.05 ± 1.33
HDL cholesterol, mmol/L	1.08 ± 0.15	0.99 ± 0.26
LDL cholesterol, mmol/L	2.65 ± 1.18	2.50 ± 0.83
BUN, mmol/L	5.32 ± 0.98	5.36 ± 1.32
CK-MB, U/L	13.47 ± 3.92	15.79 ± 14.07
Uric acid, µmol/L	330.06 ± 75.94	338.7 ± 70.41
Creatinine, µmol/L	59.92 ± 11.67	62.86 ± 10.82

Supplementary Table 2.

Baseline characteristics of the heart failure cohort stratified by TMAVA.

	**ALL**<br>(n=1647)	**Quartile 1**<br>**<=0.300µM**<br>(n=419)	**Quartile 2**<br>**0.300-0.472µM**<br>(n=406)	**Quartile 3**<br>**0.472-0.742µM**<br>(n=413)	**Quartile 4**<br>**>0.742µM**<br>(n=409)	**p Value**
**Demographic characteristics**						
Age (years)	59.1±14.3	60.1±13.1	57.8±13.8	58.5±14.7	60.5±15.2	0.001
Male (%)	66.7	63.2	67.2	67.3	69.2	0.318
Smoker (%)	38.7	37.5	28.2	39.2	39.9	0.898
Drinker (%)	23.8	23.6	23.6	22.8	25.2	0.876
SBP (mm Hg)	128 (113-144)	130 (115-147)	126 (112-143)	126 (112-144)	128 (113-142)	0.202
DBP (mm Hg)	80 (70-90)	80 (69-91)	80 (70-90)	78 (69-88)	78 (69-88)	0.128
Heart rate (beats per min)	82 (70-98)	84 (70-100)	82 (70-96)	80 (70-96)	80 (70-99)	0.536
NYHA class II/III/IV (%)	40.0/38.1/21.9	41.1/42.0/16.9	42.1/38.9/19.0	44.1/33.7/22.3	32.5/37.9/29.6	<0.001
**Medical history, n (%)**						
Hypertension	82.5	83.5	84.5	81.1	80.9	0.446
Diabetes	29.9	31.7	27.1	28.1	32.8	0.217
Dyslipidemia	20.8	23.2	20.7	19.6	19.8	0.571
Coronary artery disease	39.0	38.9	36.2	40.4	40.3	0.572
**Laboratory measurements**						
Fasting glucose (mmol/l)	5.80 (5.03-7.21)	6.06 (5.13-7.61)	5.64 (4.95-6.89)	5.67 (5.07-6.73)	5.84 (4.99-7.45)	0.017
Total cholesterol (mmol/L)	3.76 (3.16-4.53)	3.82 (3.23-4.57)	3.84 (3.22-4.55)	3.76 (3.19-4.49)	3.71 (3.03-4.54)	0.375
Triglycerides (mmol/L)	1.10 (0.79-1.61)	1.09 (0.78-1.56)	1.08 (0.79-1.62)	1.17 (0.84-1.64)	1.08 (0.79-1.62)	0.449
LDL cholesterol (mmol/L)	2.30 (1.79-2.90)	2.33 (1.84-2.85)	2.35 (1.84-2.94)	2.29 (1.75-2.93)	2.26 (1.75-2.86)	0.435
HDL cholesterol (mmol/L)	0.93 (0.76-1.13)	0.94 (0.77-1.13)	0.93 (0.77-1.14)	0.94 (0.78-1.15)	0.91 (0.72-1.11)	0.142
NT-proBNP (pg/ml)	2440 (711-6898)	2107 (665-5200)	2131 (622-5847)	2230 (636-6411)	3900 (1126-9000)	<0.001
hsCRP (mg/L)	4.60 (1.55-15.97)	5.00 (1.60-20.1)	3.80 (1.25-12.05)	3.90 (1.40-13.00)	5.80 (1.85-23.05)	0.035
eGFR (ml/min/1.73 m2)	73.4 (56.5-90.4)	78.4 (62.3-96.6)	76.4 (60.2-92.3)	73.0 (57.8-90.2)	67.0 (45.4-84.2)	<0.001
**Echocardiography**						

LVEF (%)	38 (30-56)	40 (31-56)	38 (30-57)	39 (30-60)	36 (29-53)	0.023

One-way ANOVA or Kruskal-Wallis test were used for numeric data comparison. The chi-square test was used for categorical data. P values presented are two-sided.

Supplementary Table 3.

Subgroup interaction analysis for the association between TMAVA levels and the primary outcomes.

Subgroups	Per SD of In-transformed plasma TMAVA	*P*	*P*-interaction
CHD			0.127
Yes	1.43 (1.18-1.72)	<0.001	
No	1.22 (1.09-1.36)	<0.001	
Diabetes			0.795
Yes	1.24 (1.06-1.46)	0.009	
No	1.28 (1.14-1.44)	<0.001	
Hypertension			<0.001
Yes	1.19 (1.07-1.32)	0.001	
No	1.81 (1.43-2.28)	<0.001	
EF			
<40	1.17 (1.05-1.31)	0.004	0.037
>=40	1.52 (1.23-1.89)	<0.001	

HRs and 95% CI were calculated with univariate Cox proportional hazards regression.

Supplementary Table 4.

The detailed parameters of the targeted MS instrument and LC gradient

**Characteristics**	**Value**
**MS Condition**	
Instrument	Q TRAP5500
Spray voltage (kV)	4.5 ESI+
Source temperature (℃)	600
Collision activation parameter	Medium
Curtain Gas (psi)	20
GS1 (psi)	35
GS2 (psi)	50
**LC Condition**	
Column	Luna 5u Silica 100A, 2.0*150 mm
Column Chamber T (℃)	35℃
Flow Rate	0.5mL/min
Mobile Phase A	1‰Propionic Acid 100%H2O
Mobile Phase B	1‰FA 100%MeOH
Gradient (B %)	1.00min- 2%<br>5.00min-95%<br>6.00min-95%<br>6.50min- 2%<br>7.00min-stop

Supplementary Table 5.

The internal standards were spiked in the samples and the accuracy of analytes (TMAO, TMAVA, γ-BB, TML and Carnitine) concentration was calculated.

Standard concentrations (μM)	Accuracy (%)				
	TMAO	TMAVA	γ-BB	TML	Carnitine
0.195	—	91.3	98.9	92.4	—
0.39	105.	95.0	95.5	97.6	107.
0.78	93.1	96.3	99.4	98.7	124.
1.56	100.	95.3	102.	99.9	110.
3.125	92.3	98.2	100.	98.8	97.3
6.25	108.	103.	102.	102.	104.
12.5	100.	99.3	101.	106.	96.6
25	102.	103.	99.1	100.	96.7
50	97.9	100.	104.	102.	98.8
100	100.	99.2	101.	97.9	101.

Supplementary Table 6.

Linear equation, correlation coefficient, LOD, LOQ of TMAO, TMAVA, γ-BB, TML and Carnitine.

Analytes	Linear equation	Correlation coefficient	LOD (μM)	LOQ (μM)	Linear range (μM)
TMAO	y=0.0098x+0.000135	0.9978	0.098	0.195	0.39-100
TMAVA	y=0.246x+0.000253	0.9998	0.006	0.012	0.0975-100
γ-BB	y=0.104x-4.02e-5	0.9999	0.012	0.025	0.0975-100
TML	y=0.125x+9.94e-5	0.9997	0.012	0.025	0.0975-100
Carnitine	y=0.224x+0.275	0.9999	0.012	0.025	0.39-100

Supplementary Table 7.

Method for validation of HPLC–MS/MS analysis of TMAO, TMAVA, γ-BB, TML and Carnitine: precision and recovery values at different concentrations in quality control samples.

Analytes	Spiked (μM)	Intraday (n = 6)		Interday (n = 18)	
		Recovery (%)	CV (%)	Recovery (%)	CV (%)
TMAO	0.78	107.26	7.65	104.15	8.86
	1.56	100.32	4.69	102.10	5.64
	3.125	103.89	2.46	104.05	3.63
TMAVA	0.39	98.25	2.44	98.39	3.48
	0.78	96.50	1.96	96.57	2.02
	1.56	94.98	1.63	95.16	2.16
	3.125	94.93	1.96	94.92	2.01
γ-BB	0.39	104.27	4.03	106.85	5.74
	0.78	98.93	3.40	99.29	2.59
	1.56	100.21	1.76	99.82	2.32
TML	0.78	109.90	1.05	107.67	4.98
	1.56	100.21	3.30	100.11	2.83
	3.125	97.07	1.54	97.81	2.00
Carnitine	25	107.2	3.31	106.73	3.12
	50	108.67	1.16	106.34	2.21
	100	101.83	2.61	102.06	2.44

Supplementary Table 8.

Association of plasma TMAVA concentrations with HF in higher γ-BB population. (The entire population including controls and HF patients was divided to lower and higher according γ-BB levels. Then OR was calculated according TMAVA quartiles.)

Variables	Quartiles of plasma TMAVA concentrations				*P* (Q4 vs. Q1)	Per SD of ln-transformed plasma TMAVA	*P*
	Q1	Q2	Q3	Q4			
OR (95% CI)							
Unadjusted	1 (reference)	0.951 (0.688-1.315)	1.014 (0.732-1.405)	1.583 (1.124-2.231)	0.009	1.186 (1.051-1.338)	0.006
Model 1	1 (reference)	1.009 (0.723-1.409)	1.092 (0.78-1.529)	1.668 (1.169-2.380)	0.005	1.206 (1.063-1.369)	0.004
Model 2	1 (reference)	1.040 (0.674-1.604)	1.128 (0.720-1.768)	1.604 (1.005-2.562)	0.048	1.182 (0.997-1.401)	0.054

Model 1: adjusted for sex, age.

Model 2: adjusted for sex, age, smoking, SBP, diabetes, HDL, LDL, eGFR.

The entire population including controls and HF patients was divided to lower and higher according γ-BB levels. Odds ratios (95% CIs) were calculated using logistic regression. P values presented are two-sided. Model 1 was adjusted for sex and age. Model 2 was adjusted for sex, age, smoking, SBP, diabetes, HDL, LDL and eGFR.

Supplementary Table 9.

Biological process analysis of the heart proteomics.

Term	Count	%	P-Value	Benjamini	Up/down
oxidation-reduction process	73	16.55	3.97E-28	8.44E-25	UP
metabolic process	43	9.75	3.96E-14	4.21E-11	UP
glycolytic process	14	3.17	6.58E-13	4.66E-10	UP
ATP biosynthetic process	12	2.72	9.56E-13	5.08E-10	UP
ATP metabolic process	14	3.17	3.15E-12	1.34E-09	UP
fatty acid metabolic process	21	4.76	5.91E-10	2.09E-07	UP
ATP synthesis coupled proton transport	10	2.27	1.08E-09	3.29E-07	UP
fatty acid beta-oxidation	12	2.72	3.50E-09	9.29E-07	UP
protein homotetramerization	13	2.95	1.23E-07	2.90E-05	UP
glycogen metabolic process	10	2.27	2.02E-07	4.30E-05	Down
translation	29	6.58	2.37E-07	4.58E-05	Down
carbohydrate metabolic process	20	4.54	3.47E-07	5.92E-05	Up
lipid metabolic process	31	7.03	3.63E-07	5.92E-05	Up
transport	76	17.23	5.57E-07	8.46E-05	Up
proton transport	11	2.49	7.75E-07	1.10E-04	Up
response to hypoxia	18	4.08	2.52E-06	3.20E-04	Down
mitochondrion organization	12	2.72	2.56E-06	3.20E-04	Up
cristae formation	6	1.36	7.20E-06	8.50E-04	Up
response to hormone	9	2.04	1.30E-05	1.38E-03	Up
cardiac muscle contraction	9	2.04	1.30E-05	1.38E-03	Up
muscle contraction	9	2.04	1.78E-05	1.80E-03	Up
substantia nigra development	8	1.81	2.31E-05	2.23E-03	Up
response to calcium ion	10	2.27	2.60E-05	2.40E-03	Down
tricarboxylic acid cycle	7	1.59	4.51E-05	3.93E-03	Up
response to nutrient	10	2.27	4.62E-05	3.93E-03	Up
glycogen catabolic process	5	1.13	5.34E-05	4.36E-03	Up
osteoblast differentiation	12	2.72	6.34E-05	4.98E-03	Up
blood coagulation, fibrin clot formation	4	0.91	1.19E-04	8.74E-03	Up
glycolytic process through fructose-6-phosphate	4	0.91	1.19E-04	8.74E-03	Up
response to activity	8	1.81	1.27E-04	8.96E-03	Up
response to drug	21	4.76	1.36E-04	9.30E-03	Up
regulation of the force of heart contraction	6	1.36	1.55E-04	1.03E-02	Down
glucose metabolic process	9	2.04	1.70E-04	1.10E-02	Up
protein homooligomerization	15	3.40	2.10E-04	1.31E-02	Up
platelet aggregation	7	1.59	2.20E-04	1.33E-02	Up
fibrinolysis	5	1.13	3.17E-04	1.87E-02	Up
response to hydrogen peroxide	8	1.81	3.70E-04	2.13E-02	Up
response to ethanol	11	2.49	3.94E-04	2.20E-02	Up
muscle filament sliding	4	0.91	4.03E-04	2.20E-02	Up
hemostasis	7	1.59	6.39E-04	3.33E-02	Down
long-chain fatty acid metabolic process	5	1.13	6.72E-04	3.33E-02	Up
cellular protein complex assembly	5	1.13	6.72E-04	3.33E-02	Up
acyl-CoA metabolic process	6	1.36	6.74E-04	3.33E-02	Up
translational elongation	7	1.59	7.18E-04	3.43E-02	Up
aging	13	2.95	7.27E-04	3.43E-02	Up
hydrogen peroxide metabolic process	4	0.91	9.35E-04	4.23E-02	Up
fructose 1,6-bisphosphate metabolic process	4	0.91	9.35E-04	4.23E-02	Up
response to oxidative stress	11	2.49	1.08E-03	4.76E-02	Up

An adjusted p-value is defined as the smallest significance level for which the given hypothesis would be rejected, when the entire family of tests is considered. Benjamini requests adjusted p-values by using the linear step-up method of Benjamini and Hochberg.

Supplementary Table 10.

Glycolytic enzymes involved pathway analysis in the heart proteomics.

	Accession	Description	Abbreviation	Fold (T/C)	p
glycolytic pathway	O08528	Hexokinase-2	Hk2	0.75	3.15E-02
	P12382	ATP-dependent 6-phosphofructokinase, liver type	Pfkl	0.60	1.21E-02
	P47857	ATP-dependent 6-phosphofructokinase, muscle type	Pfkm	0.56	1.76E-04
	P05064	Fructose-bisphosphate aldolase A	Aldoa	0.70	1.46E-03
	Q91Y97	Fructose-bisphosphate aldolase B	Aldob	0.64	8.97E-03
	P17751	Triosephosphate isomerase	Tpi1	0.66	1.66E-02
	P09411	Phosphoglycerate kinase 1	Pgk1	0.73	3.16E-03
	P52480	Pyruvate kinase PKM	Pkm	0.67	4.73E-05
	P16125	L-lactate dehydrogenase B chain	Ldhb	0.76	1.17E-03
	P06151	L-lactate dehydrogenase A chain	Ldha	0.46	3.16E-05
TCA cycle	Q9JK42	[Pyruvate dehydrogenase (acetyl-transferring)] kinase isozyme 2, mitochondrial	Pdk2	1.49	2.10E-02
	O70571	[Pyruvate dehydrogenase (acetyl-transferring)] kinase isozyme 4, mitochondrial	Pdk4	2.95	4.04E-04
	Q9D2G2	Dihydrolipoyllysine-residue succinyltransferase component of 2-oxoglutarate dehydrogenase complex, mitochondrial	Dlst	1.30	1.60E-02
	P14152	Malate dehydrogenase, cytoplasmic	Mdh1	0.66	4.63E-03
	Q9WUM5	Succinate--CoA ligase [ADP/GDP-forming] subunit alpha, mitochondrial	Suclg1	1.19	2.53E-02

An adjusted p-value is defined as the smallest significance level for which the given hypothesis would be rejected, when the entire family of tests is considered.

Supplementary Table 11.

Cellular component analysis of the heart proteomics.

Term	Count	%	P-Value	Benjamini
mitochondrion	205	46.49	1.54E-100	6.47E-98
extracellular exosome	230	52.15	9.13E-86	1.92E-83
mitochondrial inner membrane	92	20.86	1.31E-67	1.84E-65
myelin sheath	55	12.47	2.14E-44	2.24E-42
focal adhesion	50	11.34	3.93E-23	3.30E-21
mitochondrial matrix	35	7.94	1.77E-21	1.24E-19
cytosol	105	23.81	8.51E-21	5.11E-19
mitochondrial proton-transporting ATP synthase complex	11	2.49	9.47E-13	4.97E-11
cytoplasm	216	48.98	6.31E-12	2.95E-10

blood microparticle	21	4.76	1.41E-11	5.92E-10
cell-cell adherens junction	30	6.80	1.10E-10	4.10E-09
sarcolemma	19	4.31	1.17E-10	4.10E-09
membrane raft	27	6.12	1.88E-10	6.09E-09
mitochondrial outer membrane	20	4.54	1.31E-09	3.94E-08
mitochondrial nucleoid	12	2.72	3.64E-09	1.02E-07
Z disc	17	3.85	1.53E-08	4.02E-07
respiratory chain	12	2.72	4.90E-08	1.21E-06
membrane	209	47.39	8.11E-08	1.89E-06
intercalated disc	11	2.49	1.44E-07	3.05E-06
vesicle	18	4.08	1.45E-07	3.05E-06
extracellular matrix	24	5.44	1.84E-07	3.67E-06
melanosome	14	3.17	3.55E-07	6.78E-06
protein complex	36	8.16	5.87E-07	1.07E-05
perinuclear region of cytoplasm	36	8.16	5.25E-06	9.19E-05
mitochondrial respiratory chain complex I	9	2.04	7.94E-06	1.33E-04
extracellular vesicle	9	2.04	1.10E-05	1.77E-04
extracellular space	60	13.61	1.20E-05	1.87E-04
stress fiber	10	2.27	1.61E-05	2.41E-04
peroxisome	13	2.95	3.49E-05	5.05E-04
mitochondrial intermembrane space	10	2.27	4.03E-05	5.65E-04
proton-transporting ATP synthase complex, coupling factor F(o)	5	1.13	1.02E-04	1.29E-03
mitochondrial proton-transporting ATP synthase complex, coupling factor F(o)	5	1.13	1.02E-04	1.29E-03
proton-transporting ATP synthase complex, catalytic core F(1)	4	0.91	1.04E-04	1.29E-03
mitochondrial proton-transporting ATP synthase complex, catalytic core F(1)	4	0.91	1.04E-04	1.29E-03
fascia adherens	5	1.13	1.44E-04	1.73E-03
cell-cell junction	15	3.40	1.62E-04	1.89E-03
cell surface	30	6.80	1.70E-04	1.93E-03
pseudopodium	5	1.13	1.98E-04	2.19E-03
T-tubule	8	1.81	2.52E-04	2.69E-03
ruffle	10	2.27	2.56E-04	2.69E-03
fibrinogen complex	4	0.91	3.52E-04	3.61E-03
cell cortex	12	2.72	3.67E-04	3.67E-03
cortical cytoskeleton	6	1.36	5.47E-04	5.35E-03
immunological synapse	6	1.36	8.49E-04	8.10E-03
cell periphery	8	1.81	9.71E-04	8.81E-03
myosin complex	7	1.59	9.75E-04	8.81E-03
cytoskeleton	42	9.52	9.86E-04	8.81E-03
neuron projection	21	4.76	1.17E-03	1.02E-02
cytoplasmic vesicle membrane	10	2.27	1.40E-03	1.20E-02
eukaryotic translation elongation factor 1 complex	3	0.68	1.44E-03	1.21E-02
uropod	4	0.91	1.55E-03	1.28E-02
myofibril	6	1.36	1.80E-03	1.45E-02
caveola	8	1.81	2.96E-03	2.35E-02
mitochondrial respiratory chain complex IV	4	0.91	4.01E-03	3.12E-02
phosphopyruvate hydratase complex	3	0.68	4.67E-03	3.50E-02
oxoglutarate dehydrogenase complex	3	0.68	4.67E-03	3.50E-02
peroxisomal matrix	4	0.91	5.81E-03	4.28E-02
peroxisomal membrane	6	1.36	6.22E-03	4.50E-02
microvillus	7	1.59	6.73E-03	4.60E-02

An adjusted p-value is defined as the smallest significance level for which the given hypothesis would be rejected, when the entire family of tests is considered. Benjamini requests adjusted p-values by using the linear step-up method of Benjamini and Hochberg.

Supplementary Table 12.

Protein Functional clustering of the heart proteomics.

Annotation functional clustering		Count	P-Value	Benjamini
Annotation Cluster 1: Enrichment Score: 69.58	Mitochondrion	157	1.32E-95	2.03E-93
	Transit peptide	94	2.09E-63	2.13E-61
	transit peptide: Mitochondrion	89	6.81E-52	7.27E-49
Annotation Cluster 2: Enrichment Score: 23.59	Oxidoreductase	68	4.87E-30	2.99E-28
	oxidation-reduction process	73	3.97E-28	8.44E-25
	oxidoreductase activity	63	1.05E-22	7.41E-20
	NAD(P)-binding domain	30	2.10E-16	1.84E-13
Annotation Cluster 3: Enrichment Score: 18.68	Mitochondrion inner membrane	66	5.05E-54	3.87E-52
	Parkinson's disease	39	3.18E-22	3.42E-20
	Oxidative phosphorylation	32	1.72E-16	7.39E-15
	Huntington's disease	36	4.26E-15	1.53E-13
	Alzheimer's disease	31	1.39E-12	3.74E-11
	Respiratory chain	13	2.20E-09	4.83E-08
	Non-alcoholic fatty liver disease (NAFLD)	20	4.60E-06	6.18E-05
Annotation Cluster 4: Enrichment Score: 12.13	Carbon metabolism	32	6.23E-19	4.47E-17
	Glycolysis	14	4.64E-14	1.58E-12
	glycolytic process	14	6.58E-13	4.66E-10
	Glycolysis / Gluconeogenesis	18	1.35E-10	2.90E-09
	Biosynthesis of amino acids	16	8.35E-08	1.50E-06
Annotation Cluster 5: Enrichment Score: 8.40	NAD	27	2.26E-15	9.90E-14
	NAD binding	14	4.03E-10	7.85E-08
	nucleotide phosphate-binding region:NAD	19	1.36E-07	2.42E-05
	binding site:NAD	11	2.08E-03	8.55E-02
Annotation Cluster 6: Enrichment Score: 8.04	Fatty acid metabolism	19	3.34E-11	9.32E-10
	Fatty acid degradation	16	1.14E-10	2.72E-09
	fatty acid metabolic process	21	5.91E-10	2.09E-07
	Lipid metabolism	28	5.53E-08	9.44E-07
	lipid metabolic process	31	3.63E-07	5.92E-05
	Fatty acid metabolism	11	1.34E-05	1.52E-04
Annotation Cluster 7: Enrichment Score: 7.11	Alzheimer's disease	31	1.39E-12	3.74E-11
	Respiratory chain	13	2.20E-09	4.83E-08
	Electron transport	15	2.05E-08	3.94E-07
	respiratory chain	12	4.90E-08	1.21E-06
	Non-alcoholic fatty liver disease (NAFLD)	20	4.60E-06	6.18E-05
	mitochondrial respiratory chain complex I	9	7.94E-06	1.33E-04
	NADH dehydrogenase (ubiquinone) activity	7	1.62E-04	4.42E-03
Annotation Cluster 8: Enrichment Score: 6.63	Cardiac muscle contraction	17	1.41E-08	2.75E-07
	cytochrome-c oxidase activity	9	2.04E-07	1.60E-05
	Non-alcoholic fatty liver disease (NAFLD)	20	4.60E-06	6.18E-05
Annotation Cluster 9:	mitochondrial proton-transporting ATP synthase complex	11	9.47E-13	4.97E-11
	ATP biosynthetic process	12	9.56E-13	5.08E-10

Enrichment Score: 6.07	ATP metabolic process	14	3.15E-12	1.34E-09
	ATP synthesis	9	2.23E-11	6.84E-10
	ATP synthesis coupled proton transport	10	1.08E-09	3.29E-07
	Hydrogen ion transport	11	1.19E-08	2.43E-07
	ATPase activity	21	7.34E-08	6.50E-06
	proton-transporting ATP synthase activity, rotational mechanism	7	4.59E-07	2.96E-05
	proton transport	11	7.75E-07	1.10E-04
	CF(0)	5	6.11E-05	5.07E-04
	CF(1)	4	7.04E-05	5.69E-04
	proton-transporting ATP synthase complex, coupling factor F(o)	5	1.02E-04	1.29E-03
	mitochondrial proton-transporting ATP synthase complex, coupling factor F(o)	5	1.02E-04	1.29E-03
	proton-transporting ATP synthase complex, catalytic core F(1)	4	1.04E-04	1.29E-03
	mitochondrial proton-transporting ATP synthase complex, catalytic core F(1)	4	1.04E-04	1.29E-03
	hydrogen ion transmembrane transporter activity	6	9.01E-04	1.77E-02
	Ion transport	22	9.91E-03	5.07E-02
	proton-transporting ATPase activity, rotational mechanism	4	1.89E-02	1.65E-01
	ion transport	22	3.23E-02	5.42E-01
Annotation Cluster 10: Enrichment Score: 6.00	Mitochondrial substrate/solute carrier	12	9.13E-09	2.67E-06
	Mitochondrial carrier domain	12	9.13E-09	2.67E-06
	repeat:Solcar 1	12	2.37E-08	6.33E-06
	repeat:Solcar 2	12	2.37E-08	6.33E-06
	repeat:Solcar 3	11	1.19E-07	2.42E-05
	translation	29	2.37E-07	4.58E-05
	Mitochondrial carrier protein	7	1.38E-05	1.21E-03
	structural constituent of ribosome	17	6.46E-04	1.35E-02
	transmembrane transport	14	8.30E-02	9.32E-01

An adjusted p-value is defined as the smallest significance level for which the given hypothesis would be rejected, when the entire family of tests is considered. Benjamini requests adjusted p-values by using the linear step-up method of Benjamini and Hochberg.

Supplementary Table 13.

List of mouse primers used for RT-qPCR and their sequences

18S	Forward	GTAACCCGTTGAACCCATT
	Reverse	CCATCCAATCGGTAGTAGCG
Acadl	Forward	TCTTTTCCTCGGAGCATGACA
	Reverse	GACCTCTCTACTCACTTCTCCAG
Acadvl	Forward	CTACTGTGCTTCAGGGACAAC
	Reverse	CAAAGGACTTCGATTCTGCCC
Fasn	Forward	TTGCTGGCACTACAGAATGC
	Reverse	AACAGCCTCAGAGCGACAAT
Cpt1b	Forward	AAGAGACCCCGTAGCCATCAT
	Reverse	GACCCAAAACAGTATCCCAATCA
CPT2	Forward	GAAGAAGCTGAGCCCTGATG
	Reverse	GCCATGGTATTTGGAGCACT
CPT1a	Forward	AACCCAGTGCCTTAACGATG
	Reverse	GAACTGGTGGCCAATGAGAT
BBOX	Forward	AGAGTCCCTCTACCCAGCAG

	Reverse	TGGTCATTGGGCCATGTGAT
OCTN2	Forward	GATCCGAACACGGAATATCAGG
	Reverse	GTCCCCATGCAAGTTAGGAGT
mt-cytb	Forward	TTCTGAGGTGCCACAGTTATT
	Reverse	GAAGGAAAGGTATTAGGGCTAAA
H19	Forward	GTACCCACCTGTCGTCC
	Reverse	GTCCACGAGACCAATGACTG
