# Supplementary material for: Gut microbiota production of trimethyl-5-aminovaleric acid reduces fatty acid oxidation and accelerates cardiac hypertrophy
Source: Nat Commun. 2022 Apr 1;13:1757. doi: 10.1038/s41467-022-29060-7 (PMC8976029; doi:10.1038/s41467-022-29060-7)
Supplement: Supplementary file 2 — Reporting Summary [file 41467_2022_29060_MOESM2_ESM.pdf]

nature research

Corresponding author(s): Lemin Zheng

Last updated by author(s): Mar 8, 2022

Reporting Summary

Nature Research wishes to improve the reproducibility of the work that we publish. This form provides structure for consistency and transparency in reporting. For further information on Nature Research policies, see Authors & Referees and the Editorial Policy Checklist.

Statistics

For all statistical analyses, confirm that the following items are present in the figure legend, table legend, main text, or Methods section.

n/a	Confirmed	
☐	☒	The exact sample size (*n*) for each experimental group/condition, given as a discrete number and unit of measurement
☐	☒	A statement on whether measurements were taken from distinct samples or whether the same sample was measured repeatedly
☐	☒	The statistical test(s) used AND whether they are one- or two-sided<br>*Only common tests should be described solely by name; describe more complex techniques in the Methods section.*
☒	☐	A description of all covariates tested
☐	☒	A description of any assumptions or corrections, such as tests of normality and adjustment for multiple comparisons
☐	☒	A full description of the statistical parameters including central tendency (e.g. means) or other basic estimates (e.g. regression coefficient) AND variation (e.g. standard deviation) or associated estimates of uncertainty (e.g. confidence intervals)
☒	☐	For null hypothesis testing, the test statistic (e.g. *F*, *t*, *r*) with confidence intervals, effect sizes, degrees of freedom and *P* value noted<br>*Give P values as exact values whenever suitable.*
☒	☐	For Bayesian analysis, information on the choice of priors and Markov chain Monte Carlo settings
☒	☐	For hierarchical and complex designs, identification of the appropriate level for tests and full reporting of outcomes
☒	☐	Estimates of effect sizes (e.g. Cohen's *d*, Pearson's *r*), indicating how they were calculated

Our web collection on statistics for biologists contains articles on many of the points above.

Software and code

Policy information about availability of computer code

Data collection	Mass spectrometer: Q Exactive Orbitrap mass spectrometer (Thermo Fisher)<br>Mass spectrometer: API 5500Q-TRAP mass spectrometer (AB SCIEX)
Data analysis	Metabolites Identification: Tracefinder 3.2(Thermo Fisher); METLIN database (Tautenhahn et al., 2012; https://metlin.scripps.edu/)<br>Metabolites pathway analysis: Metaboanalyst (Chong et al., 2018; http://www.metaboanalyst.ca/MetaboAnalyst/)<br>Protein Identification: Proteome Discoverer 1.4 (Thermo Fisher); mouse Uniprot database (version 2021_02)(UniProt Consortium, 2018; https://www.uniprot.org/)<br>Protein function analysis: DAVID (Dennis et al., 2003; https://david.ncifcrf.gov/)<br>GraphPad Prism, 7

For manuscripts utilizing custom algorithms or software that are central to the research but not yet described in published literature, software must be made available to editors/reviewers. We strongly encourage code deposition in a community repository (e.g. GitHub). See the Nature Research guidelines for submitting code & software for further information.

Data

Policy information about availability of data

All manuscripts must include a data availability statement. This statement should provide the following information, where applicable:

- Accession codes, unique identifiers, or web links for publicly available datasets
- A list of figures that have associated raw data
- A description of any restrictions on data availability

The raw proteomics MS data generated in this study have been deposited to the ProteomeXchange Consortium [http://proteomecentral.proteomexchange.org] via the iProX partner repository under accession code PXD028230. [http://proteomecentral.proteomexchange.org/cgi/GetDataset?ID=PXD028230]. The raw metabolomics MS data generated in this study have been deposited in the MetaboLights database under accession code MTBLS3174 [www.ebi.ac.uk/metabolights/MTBLS3174]. Experimental data that support the findings of this study are available from the corresponding authors on reasonable request. Source data are

provided with this paper.

Field-specific reporting

Please select the one below that is the best fit for your research. If you are not sure, read the appropriate sections before making your selection.

☒ Life sciences ☐ Behavioural & social sciences ☐ Ecological, evolutionary & environmental sciences

For a reference copy of the document with all sections, see nature.com/documents/nr-reporting-summary-flat.pdf

Life sciences study design

All studies must disclose on these points even when the disclosure is negative.

Sample size	Sample sizes were predetermined based on effect size, standard deviation, and significance level required to attain statistical significance of p<0.05 with a 90% probability on the basis of previous experiments using similar methodologies and were deemed sufficient to account for any biological/technical variability.
Data exclusions	Outliers were excluded.
Replication	In vivo experiments were carried out on multiple independent cohorts of animals with similar results. Proteomic data were further followed up by alternative approaches, such as RT-qPCR.
Randomization	For in vivo studies, mice in each genotype were randomly assigned to treatment groups.
Blinding	Investigators were not blinded during animal experiments but were blinded in human study.

Reporting for specific materials, systems and methods

We require information from authors about some types of materials, experimental systems and methods used in many studies. Here, indicate whether each material, system or method listed is relevant to your study. If you are not sure if a list item applies to your research, read the appropriate section before selecting a response.

Materials & experimental systems

n/a	Involved in the study	
☐	☒	Antibodies
☒	☐	Eukaryotic cell lines
☒	☐	Palaeontology
☐	☒	Animals and other organisms
☐	☒	Human research participants
☒	☐	Clinical data

Methods

n/a	Involved in the study	
☒	☐	ChIP-seq
☒	☐	Flow cytometry
☒	☐	MRI-based neuroimaging

Antibodies

Antibodies used	All antibodies were used according to the supplier's instructions.<br>Alpha Tubulin (Proteintech; Cat: 66031-1-Ig)<br>SDHB (GeneTex; Cat: GTX113833)<br>ATP5A1 (GeneTex; Cat: GTX101741)<br>UQCRC2 (GeneTex; Cat: GTX114873)<br>NDUFB8 (Abcam; Cat: ab192878)
Validation	Alpha Tubulin: http://www.ptgcn.com/products/tubulin-Alpha-Antibody-66031-1-Ig.htm<br>SDHB: http://wws.genetex.com/SDHB-antibody-GTX113833.html<br>ATP5A1: http://wws.genetex.com/ATP5A1-antibody-C2C3-C-term-GTX101741.html<br>UQCRC2: http://wws.genetex.com/ATP5A1-antibody-C2C3-C-term-GTX101741.html<br>NDUFB8: https://www.abcam.cn/ndufb8-antibody-epr15961-ab192878.html

Animals and other organisms

Policy information about studies involving animals; ARRIVE guidelines recommended for reporting animal research

Laboratory animals	Mice were housed at 22°C under a 12 hr light/dark cycle with free access to food and water. BBOX knockout mice (BBOX-/-) were created using CRISPR-Cas9 in C57BL6/J background. Five base pairs were deleted after AATGACCA in the BBOX, exon 2. All experiments used age-matched male littermates and were conducted at 22°C except the cold exposure test at 4°C. All protocols

	for mouse experiments were approved by the Ethics Committee of Animal Research, Peking University Health Science Center.
Wild animals	The study did not involve wild animals.
Field-collected samples	The study did not involve samples collected from the field.
Ethics oversight	All protocols for mouse experiments were approved by the Ethics Committee of Animal Research, Peking University Health Science Center and the Animal Care and Ethics Committee of Tongji Medical College of Huazhong University of Science and Technology.

Note that full information on the approval of the study protocol must also be provided in the manuscript.

Human research participants

Policy information about studies involving human research participants

Population characteristics	The learning cohort leading to the discovery of TMAVA was described previously in our recent publication[Zhao, M. et al. TMAVA, a metabolite of intestinal microbes, is increased in plasma from patients with liver steatosis, inhibits γ-butyrobetaine hydroxylase, and exacerbates fatty liver in mice. Gastroenterology 158, 2266–2281.e2227 (2020).]. TMAVA levels in patients with hypertension in that cohort were analyzed in this new study. The identification of hypertensive patients was based on a clearly documented medical history of hypertension with a systolic blood pressure≥140 mm Hg or a diastolic blood pressure≥90 mm Hg. The baseline characteristics of subjects in the learning cohort are presented in Supplementary Table 1 in the manuscript. The validation cohort, on the other hand, is a prospective population-based cohort study. At recruitment, 1647 participants with heart failure were enrolled between 2008-2017, corresponding to the longest 84-month follow-up visit (Supplementary Table 2 in the manuscript). Follow-up for mortality and specific outcome of adverse events were implemented by trained interviewers. The participants were tracked until the first occurrence of defined outcomes, including cardiac death and heart transplantation. All blood samples were collected at the fasting state, and stored at -80 °C immediately until analysis.
Recruitment	Inclusion and diagnosis of these patients were assessed by cardiologists from the Tongji Hospital Affiliated to Tongji Medical College, Huazhong University of Science and Technology, Wuhan, China. Indications for the enrolment of HF and exclusion criteria have been reported previously[Huang, J. et al. ADRB2 polymorphism Arg16Gly modifies the natural outcome of heart failure and dictates therapeutic response to beta-blockers in patients with heart failure. Cell Discov. 4, 57 (2018).]. Briefly, subjects with clinically significant valvar heart disease, acute myocardial infarction or unstable angina within one month, as well as those with malignant tumor, were excluded from the study. Patients with severe coronary heart disease without complete revascularization therapy were not specifically excluded. Concomitantly, control subjects without significant cardiac disease consecutively screened with coronary angiogram and echocardiography were selected at random in the same hospital from October 2013 to March 2017.
Ethics oversight	The study was designed and carried out in accordance with the principles of the declaration of Helsinki and approved by the Ethics Committee of Tongji Medical College. All subjects provided informed consent.

Note that full information on the approval of the study protocol must also be provided in the manuscript.
